# Quantifying and predicting antimicrobials and antimicrobial resistance genes in waterbodies through a holistic approach: a study in Minnesota, United States

**DOI:** 10.1038/s41598-021-98300-5

**Published:** 2021-09-21

**Authors:** Irene Bueno, Amanda Beaudoin, William A. Arnold, Taegyu Kim, Lara E. Frankson, Timothy M. LaPara, Kaushi Kanankege, Kristine H. Wammer, Randall S. Singer

**Affiliations:** 1 Department of Veterinary and Biomedical Sciences, College of Veterinary Medicine, University of Minnesota, 1971 Commonwealth Ave., St. Paul, MN 55108 USA; 2 Minnesota Department of Health, P.O. Box 64975, St. Paul, MN 55164-0975 USA; 3 Department of Civil, Environmental, and Geo-Engineering, University of Minnesota, 500 Pillsbury Dr. SE, Minneapolis, MN 55455 USA; 4 Water Resources Science Program, University of Minnesota, 1985 Buford Ave., St. Paul, MN 55108 USA; 5 Center for Animal Health and Food Safety, College of Veterinary Medicine, University of Minnesota, 1354 Eckles Ave., St. Paul, MN 55108 USA; 6 College of Arts & Sciences, University of St. Thomas, 2115 Summit Ave., St. Paul, MN 55105 USA

**Keywords:** Antimicrobial resistance, Environmental monitoring

## Abstract

The environment plays a key role in the spread and persistence of antimicrobial resistance (AMR). Antimicrobials and antimicrobial resistance genes (ARG) are released into the environment from sources such as wastewater treatment plants, and animal farms. This study describes an approach guided by spatial mapping to quantify and predict antimicrobials and ARG in Minnesota’s waterbodies in water and sediment at two spatial scales: macro, throughout the state, and micro, in specific waterbodies. At the macroscale, the highest concentrations across all antimicrobial classes were found near populated areas. Kernel interpolation provided an approximation of antimicrobial concentrations and ARG abundance at unsampled locations. However, there was high uncertainty in these predictions, due in part to low study power and large distances between sites. At the microscale, wastewater treatment plants had an effect on ARG abundance (*sul1* and *sul2* in water; *bla*_SHV_, *intl1*, *mex*B, and *sul2* in sediment), but not on antimicrobial concentrations. Results from sediment reflected a long-term history, while water reflected a more transient record of antimicrobials and ARG. This study highlights the value of using spatial analyses, different spatial scales, and sampling matrices, to design an environmental monitoring approach to advance our understanding of AMR persistence and dissemination.

## Introduction

The role that the natural environment plays in the dissemination and persistence of antimicrobial resistance (AMR) has been increasingly recognized in recent years^1,2^. Antimicrobials and antimicrobial resistance genes (ARG) can enter the environment from several anthropogenic point sources, including pharmaceutical factories, hospitals, wastewater treatment plants, animal husbandry operations, and ethanol plants^3–5^. Much of this discharge ends up in surface water (e.g., rivers and lakes), making the aquatic environment an important recipient of antimicrobials, antimicrobial resistant bacteria, and ARG^6,7^.

Once in the aquatic environment, antimicrobials, antimicrobial resistant bacteria, and ARG can further disseminate. ARG in particular can be exchanged between non-pathogenic environmental bacteria and pathogenic bacteria mainly through horizontal gene transfer (HGT)^8,9^. Some studies have illustrated the potential for specific genes to transfer to pathogenic bacteria. For example, the plasmid-encoded *qnr*A genes, which confer quinolone resistance and are clinically relevant, are thought to originate in *Shewanella algae*, an aquatic Gram negative bacterium^10^. Other studies have examined the breadth of ARG diversity in the aquatic environment, often focusing on genes that have the potential to be exchanged between bacteria. For example, Zhang et al. (2009) conducted a comprehensive review on HGT across different gene families^11^. Despite an increasing number of published studies evaluating antimicrobials and ARG in the natural environment (rivers, lakes, soil, air, and wildlife), the ability to associate environmental findings with specific sources of antimicrobials and ARG, and to attribute environmental findings to specific health risks in humans and animals, is still lacking^4^.

The importance of monitoring AMR in the natural environment has been recognized internationally by the World Health Organization (WHO), Food Agricultural Organization (FAO), and the Office International des Epizooties, a.k.a. World Organisation for Animal Health (OIE). The National Antimicrobial Resistance Monitoring System (NARMS) within the United States (U.S.), which has historically focused on collection of bacterial isolates from human clinical specimens, retail meats, and healthy food animals, is currently discussing an expansion to include surveillance for bacterial isolates in environmental samples^12^. In addition, multidrug resistance (MDR) has increased globally, becoming a public health threat. Several recent studies have revealed that the emergence of multidrug-resistant bacterial pathogens have different origins, including the natural environment^13^. Therefore, initiatives that collect data from humans, animals, and the natural environment are essential to understand the dissemination and persistence of AMR, including MDR, and its impact on animal, ecosystem, and public health.

Geospatial approaches play an important role to map antimicrobial and AMR sources, to evaluate their persistence and dissemination in the natural environment at different spatial scales, and to guide environmental sampling and monitoring^14^. The usefulness of these approaches has been demonstrated by other researchers. For example, Pruden et al. (2011) quantified the relationship between selected ARG and landscape features^15^; de la Torre et al. (2012) built a risk model of European soil vulnerability to different antibiotics^16^; and Singer et al. (2019) used antibiotic prescribing data to model the most likely areas in a watershed for antibiotics to accumulate^17^.

In this study, our goal was to describe an approach guided by spatial mapping to quantify and predict antimicrobials and ARG in waterbodies at two spatial scales (macro and micro). This approach can be useful when designing environmental monitoring of AMR. We evaluated antimicrobial compounds and ARG in two environmental matrices (water and sediment) collected from waterbodies in Minnesota, United States.

## Results

The environmental dimension of antimicrobial residues and ARG can be described at two different spatial scales: a macroscale encompassing input sources of antimicrobials and AMR throughout the state of Minnesota, and a microscale, comprising the distribution of antimicrobials and ARG in specific waterbodies in relation to their proximate input sources.

### Macroscale analysis

**Figure 1:**
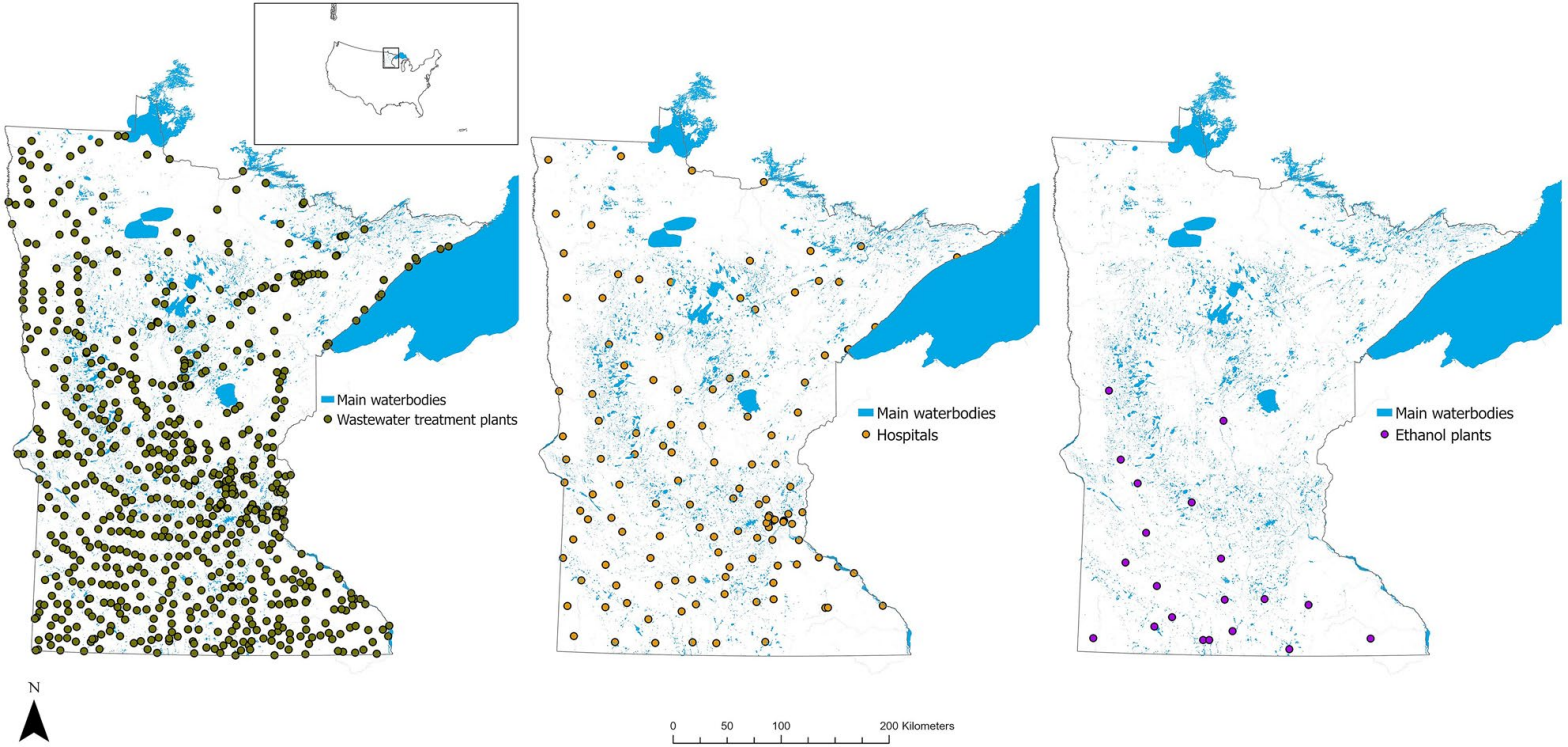
Point map of locations for wastewater treatment plants, hospitals, and ethanol plants in Minnesota.

**Figure 2:**
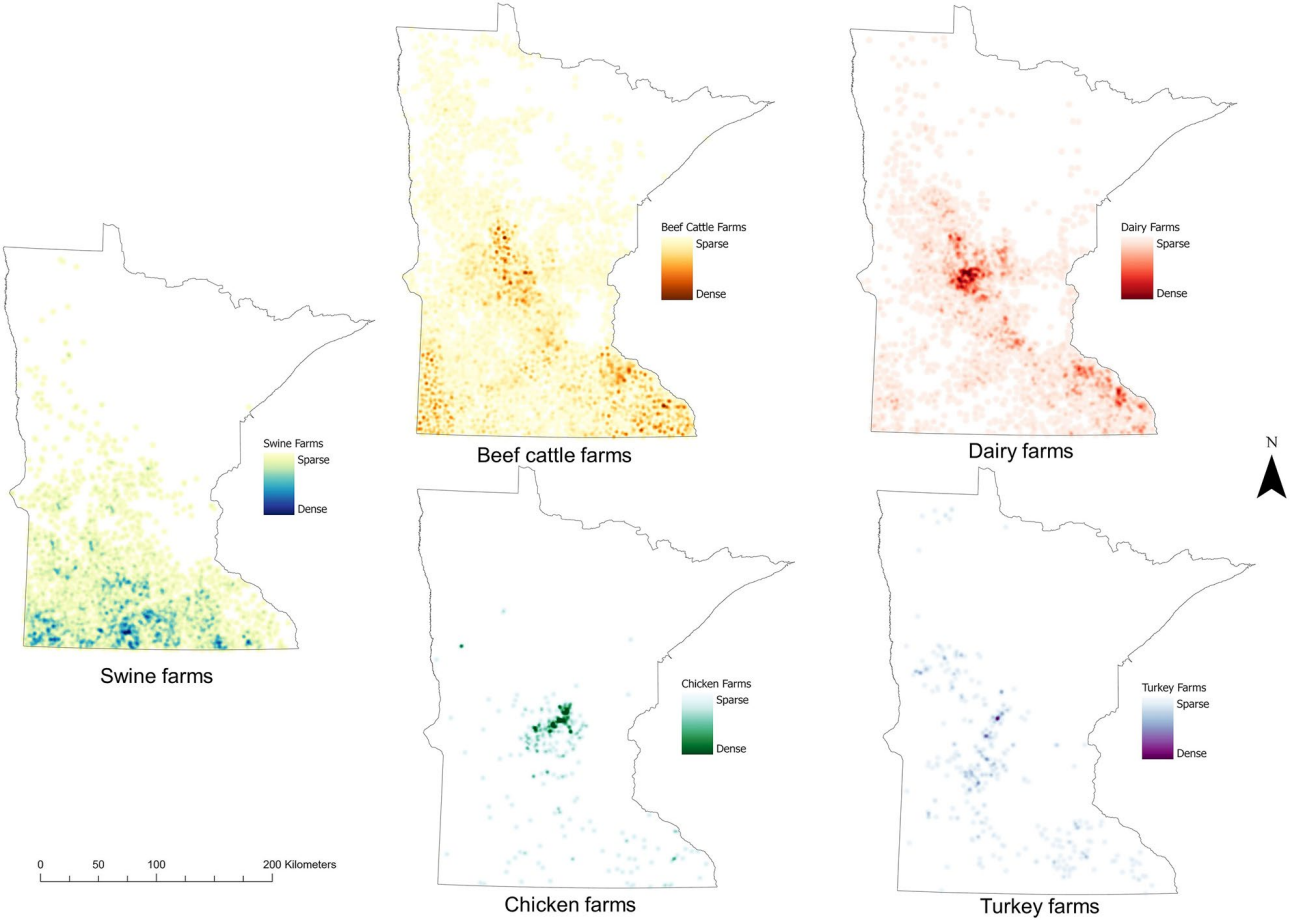
Heat maps of farm density for the different livestock species in Minnesota.

Potential input sources of antimicrobials and ARG were located heterogeneously throughout the state. Human population density was highest in the eastern-central part of Minnesota, which includes the Twin Cities metropolitan area. Wastewater treatment plants (n = 715) and hospitals (n = 128) were located throughout the state, and ethanol plants (n = 20) were largely located in the southern area. Regional livestock density varied by species. Swine farm density was highest in the southern part of the state, especially the south-western and south-central area. Cattle farms were located throughout the state, but the highest density of farms was found in the south-west (beef cattle) and central and south-east regions (dairy). Most turkey and chicken farms were in the eastern (turkey) and central (chicken) regions of Minnesota (Figs. 1, 2).

**Figure 3:**
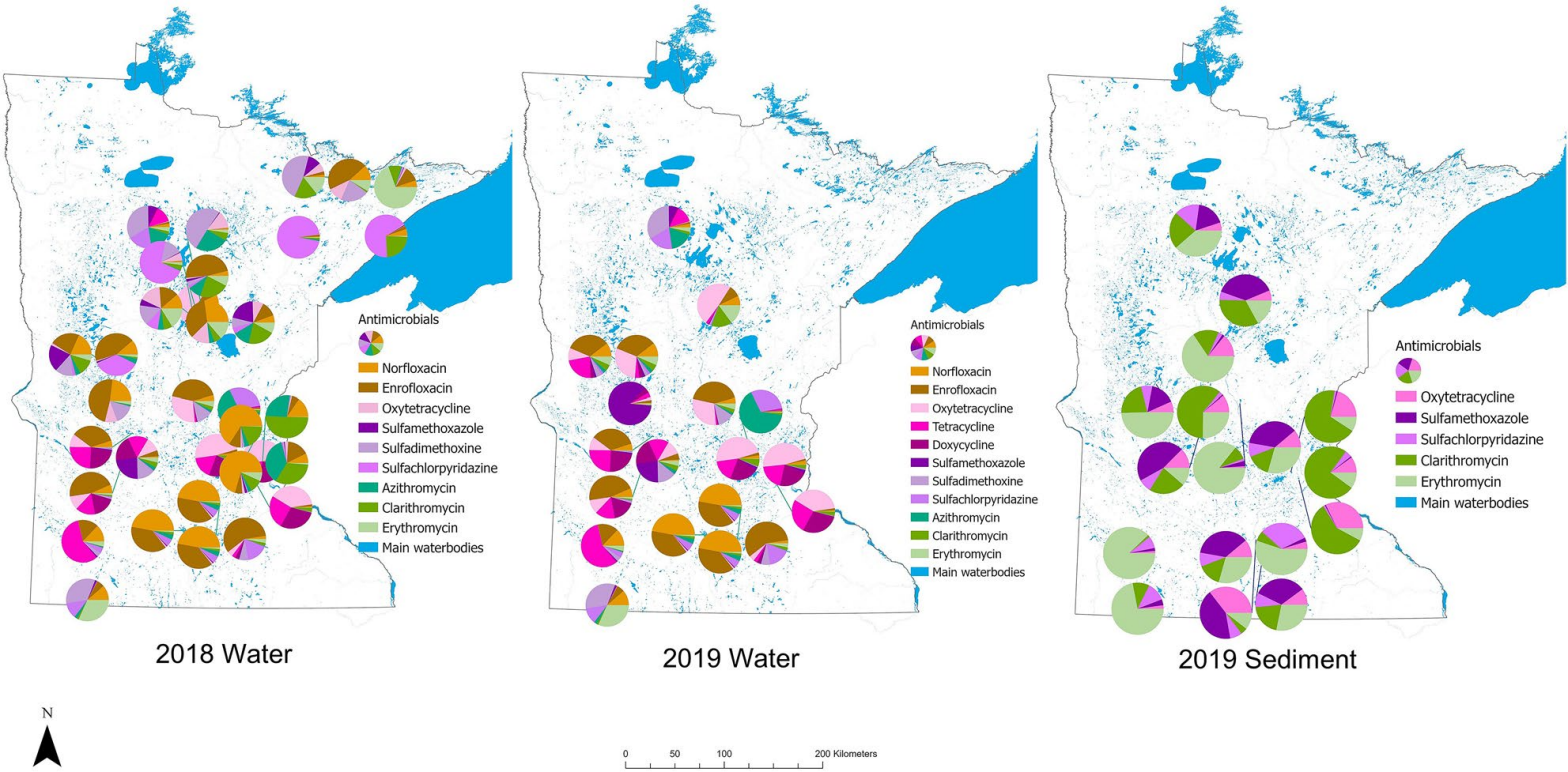
Antimicrobial concentrations for main antimicrobials detected in sampling sites in 2018 (water) and 2019 (water and sediment).

**Figure 4:**
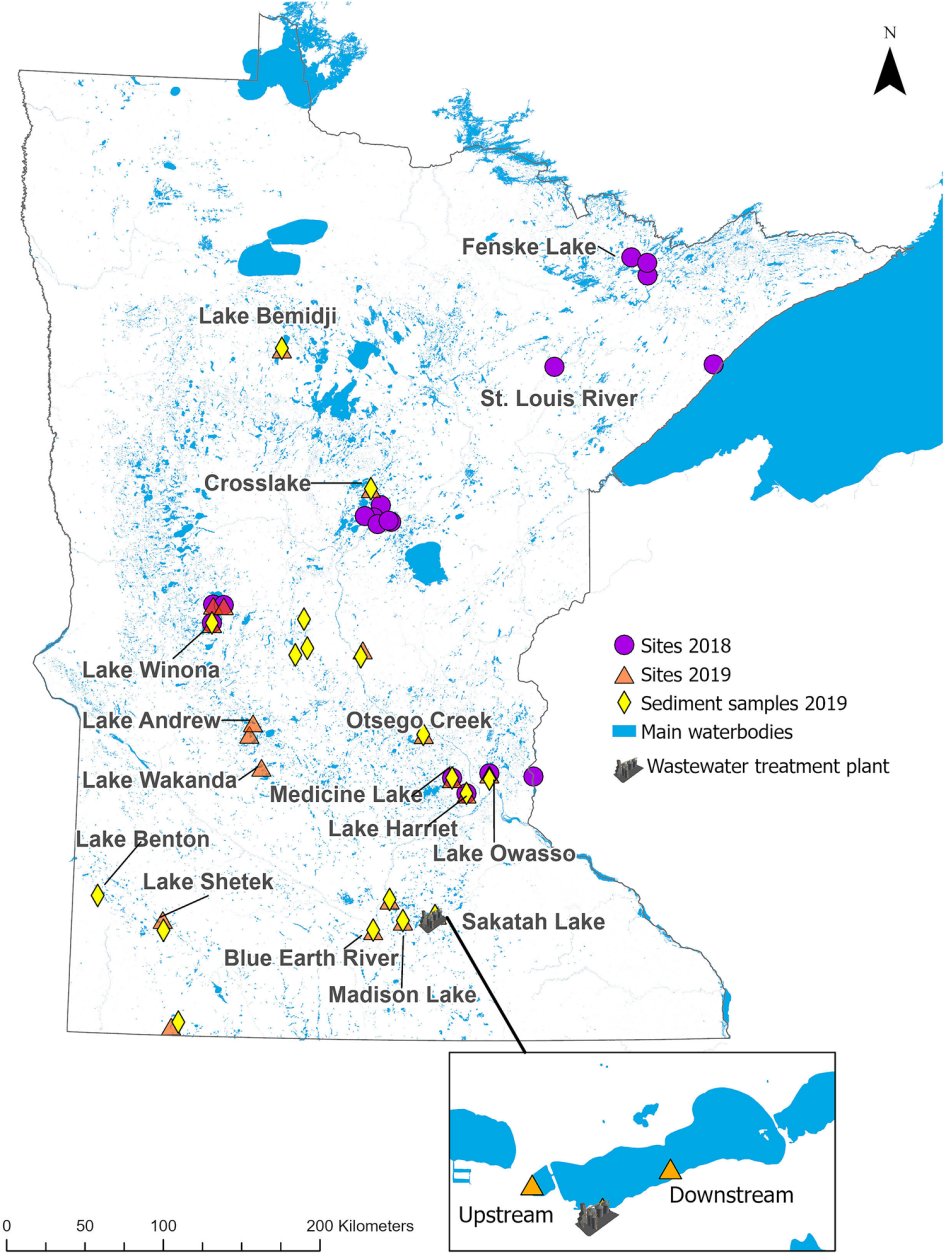
Map of Minnesota with sampling sites for each one of the sample types. The map shows the sampling at the macroscale, and an example of one of the sites with influence of a wastewater treatment plant where there was sampling conducted at the microscale. This figure also captures the names of the sites with highest antimicrobial concentrations and ARG abundance.

Sampling locations with the highest antimicrobial concentrations in water samples in 2018 were: St. Louis River (sulfachloropyridazine: 476.6 ng/L), Lake Harriet (norfloxacin: 252.7 ng/L), Medicine Lake (norfloxacin: 234.0 ng/L), Lake Owasso (clarithromycin: 204.8 ng/L), and Lake Winona (enrofloxacin: 188.0 ng/L). In 2019, sites with the highest antimicrobial concentrations in water samples were: Lake Winona (sulfamethoxazole: 445.0 ng/L), Lake Shetek (tetracycline: 288.5 ng/L), Lake Owasso (oxytetracycline: 288.1 ng/L), Lake Harriet (oxytetracycline: 220.0 ng/L), and Otsego Creek (azithromycin: 208.2 ng/L). The sampling sites with the highest antimicrobial concentrations in sediment (2019 data only) were: Lake Shetek (erythromycin: 1834.3 ng/g), Medicine Lake (sulfadimethoxine: 733.0 ng/g), and Madison Lake (tetracycline: 390.0 ng/g and doxycycline: 380.0 ng/g) (Figs. 3, 4). Summary statistics for all antimicrobials analyzed across sites in water and sediment samples in 2018 and 2019 can be found in Tables 1, 2 and 3.

**Table 1:** Summary statistics (arithmetic mean ± standard error, standard deviation (SD), median, and range: minimum (min) and maximum (max)) for antimicrobial concentrations (ng/L) in water samples across sites analyzed in 2018 (n = 17).

Antimicrobial	Mean ± SE	SD	Median	Range: min, max
Norfloxacin	42.5 ± 19.4	79.9	4.0	1.2, 252.7
Ciprofloxacin	6.2 ± 3.1	12.7	1.2	0.0, 50.9
Enrofloxacin	30.8 ± 13.8	57.0	7.50	0.41, 188.0
Ofloxacin	7.9 ± 3.4	14.1	1.6	0.1, 50.2
Oxytetracycline	8.6 ± 3.2	12.9	1.8	0.1, 43.2
Chlortetracycline	46.5 ± 12.6	51.9	21.6	0.8, 174.8
Erythromycin	10.1 ± 3.6	14.8	2.8	0.6, 45.4
Roxythromycin	0.3 ± 0.0	0.1	0.3	0.1, 0.5
Tylosin	10.9 ± 9.3	38.4	1.1	0.0, 159.5
Azithromycin	11.9 ± 6.4	26.3	2.7	0.1, 108.7
Clarithromycin	25.5 ± 12.0	49.5	6.7	0.7, 204.8
Trimethropin	5.9 ± 3.3	13.5	1.2	0.2, 56.8
Lincomycin	18.8 ± 5.3	21.9	8.5	0.9, 71.3
Sulfapyridine	8.7 ± 3.53	14.6	1.7	0.4, 46.3
Sulfadiazine	3.1 ± 1.5	6.3	0.4	0.1, 25.9
Sulfamethoxazole	11.0 ± 8.1	33.4	0.8	0.3, 138.2
Sulfamethazine	8.9 ± 3.6	14.6	0.5	0.1, 45.9
Sulfachlorpyridazine	39.1 ± 28.2	116.1	0.6	0.4, 476.6
Sulfadimethoxine	21.5 ± 8.9	36.1	2.0	0.4, 123.0

**Table 2:** Summary statistics (arithmetic mean ± standard error, standard deviation (SD), median, and range: minimum (min) and maximum (max)) for antimicrobial concentrations analyzed in water samples (ng/L) across sites in 2019 (n = 19).

Antimicrobial	Mean ± SE	SD	Median	Range: min, max
Ciprofloxacin	16.4 ± 5.24	22.9	3.5	1.5, 76.5
Norfloxacin	16.9 ± 4.6	19.9	4.4	0.7, 64.9
Enrofloxacin	36.8 ± 12.8	55.9	10.0	0.9, 185.9
Ofloxacin	12.3 ± 4.1	18.0	2.1	0.6, 63.7
Doxycycline	33.7 ± 12.3	53.5	0.9	0.0, 162.4
Tetracycline	46.7 ± 17.5	76.1	6.8	0.3, 288.5
Oxytetracycline	45.4 ± 19.7	85.7	8.9	0.1, 288.1
Chlortetracycline	2.6 ± 0.8	3.3	1.3	0.3, 14.2
Erythromycin	5.9 ± 2.6	11.2	2.0	1.1, 48.6
Roxithromycin	1.8 ± 0.4	1.7	1.2	0.0, 6.9
Tylosin	28.1 ± 11.2	48.9	1.9	0.2, 155.9
Azithromycin	13.1 ± 10.9	47.3	0.8	0.1, 208.2
Clarithromycin	5.2 ± 1.6	6.9	1.7	0.7, 22.1
Carbadox	1.2 ± 0.5	2.2	0.4	0.0, 7.2
Trimethoprim	11.3 ± 3.0	13.1	10.2	0.3, 42.7
Lincomycin	1.1 ± 0.3	1.1	0.5	0.0, 4.0
Amoxicillin	0.3 ± 0.1	0.6	0.1	0.0, 2.4
Penicillin V	1.2 ± 0.6	2.6	0.1	0.0, 8.5
Penicillin G	4.2 ± 2.0	8.8	0.1	0.0, 32.7
Sulfadimethoxine	7.5 ± 3.0	13.2	1.4	0.0, 51.2
Sulfapyridine	12.0 ± 4.4	19.0	1.8	0.8, 63.1
Sulfadiazine	6.1 ± 3.3	14.4	0.8	0.1, 51.4
Sulfamethoxazole	28.6 ± 23.3	101.5	1.1	0.1, 444.9
Sulfamethazine	0.9 ± 0.3	1.3	0.2	0.0, 4.4
Sulfachlorpyridazine	9.8 ± 4.9	21.7	3.2	0.1, 95.3

**Table 3:** Summary statistics (arithmetic mean ± standard error, standard deviation (SD), median, and range: minimum (min) and maximum (max)) for antimicrobial concentrations analyzed in sediment samples (ng/g) across sites in 2019 (n = 17).

Antimicrobial	Mean ± SE	SD	Median	Range: min, max
Tetracycline	36.8 ± 22.3	92.1	7.5	3.9, 390.0
Oxytetracycline	18.7 ± 3.7	15.1	14.5	2.9, 59.0
Doxycycline	59.5 ± 23.8	97.9	14.6	0.9, 380.0
Erythromycin	226.2 ± 116.3	479.6	23.1	13.1, 1834.3
Roxithromycin	174.3 ± 75.7	312.0	21.6	4.6, 967.0
Tylosin	29.8 ± 9.1	37.4	16.9	5.2, 142.1
Clarithromycin	50.7 ± 11.7	48.2	26.0	5.2, 162.9
Carbadox	0.6 ± 0.3	1.4	0.1	0.0, 5.8
Trimethoprim	9.6 ± 3.9	16.1	1.0	0.1, 48.0
Sulfadimethoxine	160.2 ± 57.6	237.6	11.5	4.8, 733.0
Sulfapyridine	51.4 ± 17.2	70.9	23.6	2.8, 243.5
Sulfadiazine	7.8 ± 1.2	4.9	8.0	0.4, 15.0
Sulfamethoxazole	21.7 ± 4.5	18.7	20.9	1.2, 52.4
Sulfamethazine	35.5 ± 20.9	86.0	5.4	0.1, 283.0
Sulfachlorpyridazine	26.2 ± 11.0	45.4	6.8	1.9, 156.1

**Figure 5:**
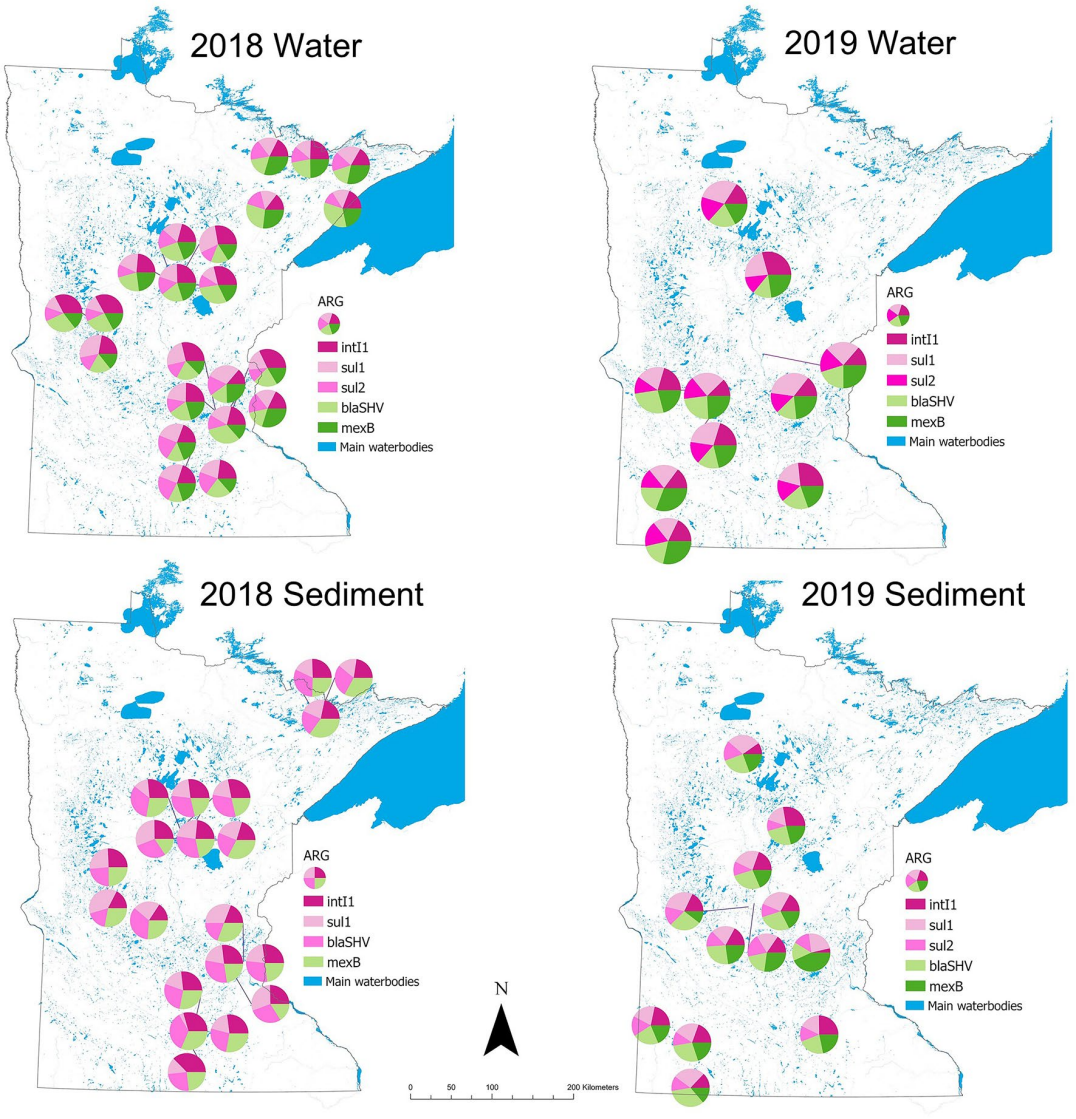
Antimicrobial resistance gene (ARG) abundance for main genes detected in sampling sites in 2018 (water and sediment) and 2019 (water and sediment).

Target genes that had ≥ 80% of non-detects across samples were removed from further analyses, following previously published approaches^18,19^. After removing those target genes with ≥ 80% of non-detects across all samples (Supplementary Tables S1 and S2), a total of 10 ARG and two integrase genes (*intI1* and *intI3*) from 2018 water samples were kept for analyses. These genes encode resistance to beta-lactamases (*bla*_OXA*,*_* bla*_SHV_, IMP-13)*,* quaternary ammonium compounds (*qacF*)*,* streptomycin (*strB*)*,* sulfonamides (*sul1, sul2, sul3*)*,* tetracyclines (*tet*(A)), or are multidrug resistance proteins (*mexB*). For 2019 water samples, seven ARG (*bla*_SHV_, *IMP-13*, *mexB, strB*, *sul1*, *sul2*, and *tet*(A)) and two integrase genes (*intI1* and *intI3*) were analyzed. For 2018 sediment samples, six ARG (*bla*_SHV,_ *ermB*, *mexB*, *strB*, *sul1*, and *tet*(A)) and one integrase gene (*intI1*) were analyzed. Lastly, for 2019 sediment samples, 10 ARG (*bla*_SHV_, *ermB*, *mefE*, *mexB*, *strB*, *sul1*, *sul2*, *sul3*, *tet*(A), and *tet*(W)) and two integrase genes (*intl1* and *intl3*) were analyzed. Locations across the state with the highest gene abundance in 2018 water samples were: Lake Winona (*sul1*: 5.9 log_10_ copies/L), Medicine Lake (*sul1*: 4.6 log_10_ copies/L, *intI1*: 5.3 log_10_ copies/L), and Blue Earth River (*sul1*: 4.4 log_10_ copies/L, *sul2*: 4.3 log_10_ copies/L) while in 2019 were Otsego Creek (*sul1*: 3.7 log_10_ copies/L), Sakatah Lake (*intI1*: 3.5 log_10_ copies/L), Lake Bemidji (*sul1*: 3.4 log_10_ copies/L), Lake Andrew (*bla*_SHV_: 3.4 log_10_ copies/L), and Crosslake (*intI1*: 3.1 log_10_ copies/L). Locations across the state with the highest gene abundance in 2018 sediment samples were: Blue Earth River (*intI1*: 4.5 log_10_ copies/g), Lake Wakanda (*bla*_SHV:_ 4.3 log_10_ copies/g), and Fenske Lake (*bla*_SHV_: 4.3 log_10_ copies/g), and in 2019 sediment samples were: Lake Benton (*bla*_SHV_: 5.5 log_10_ copies/g, *intI1*: 5.4 log_10_ copies/g, *sul1*: 5.1 log_10_ copies/g), Otsego Creek (*mexB*: 4.6 log_10_ copies/g), and Lake Sakatah (*intI1*: 4.2 log_10_ copies/g). (Figs. 4, 5). Summary statistics for all ARG analyzed across sites in water and sediment samples in 2018 and 2019 can be found in Supplementary Tables S3–S6 and ARG abundance quantification can be found in Supplementary Figures S1–S4.

**Figure 6:**
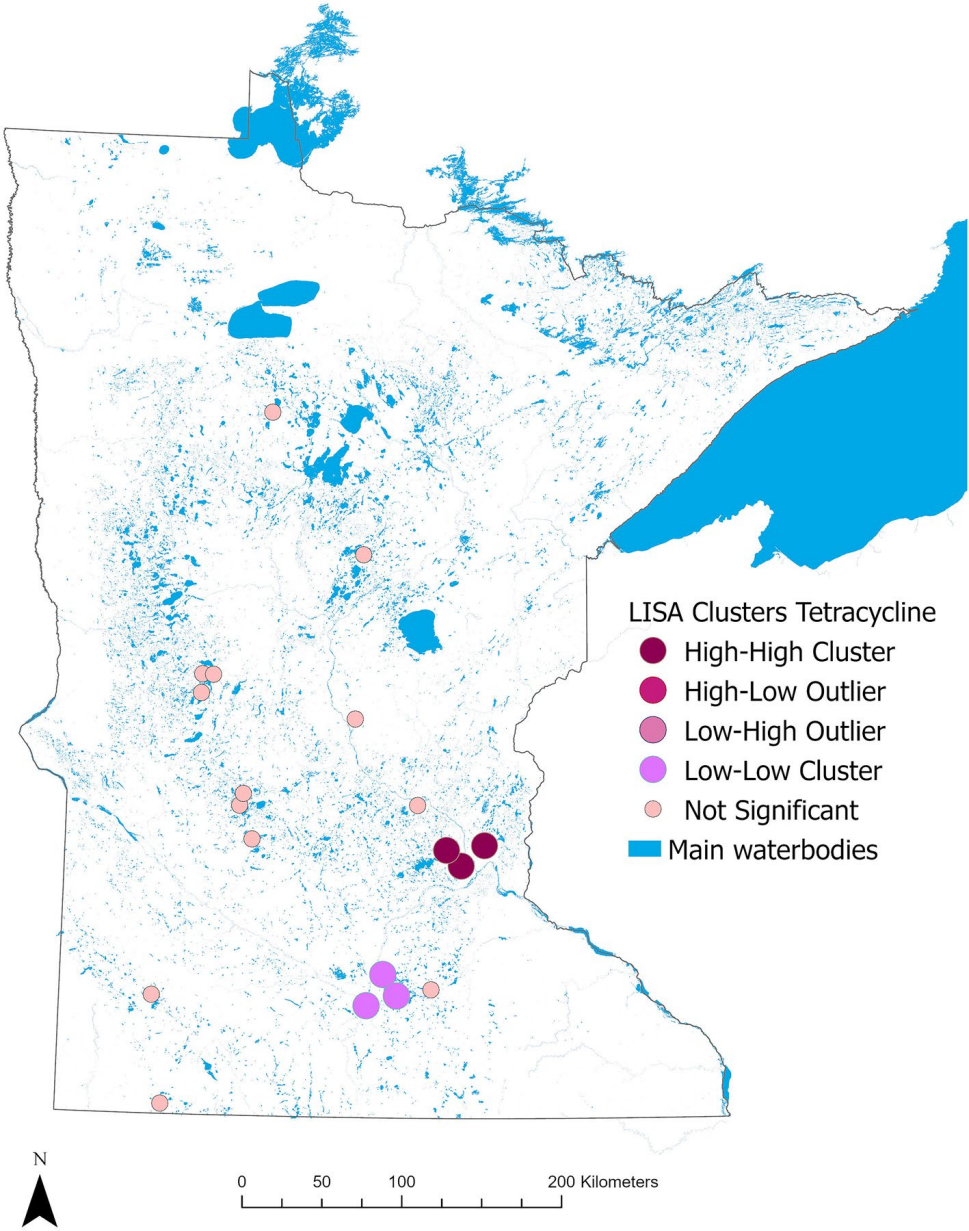
Results from the Anselin’s Local Morans I (LISA) test for tetracycline concentrations in water samples showing two significant clusters: one high–high, and one low–low in two areas.

The Global Moran’s I test showed a statistically significant positive spatial autocorrelation for tetracycline concentrations in water (Index = 0.50, z-score = 2.30, *p* = 0.02), indicating a general tendency for locations near each other (distance between these sites was on average 15 km) to have similar tetracycline concentration values, while the concentrations of ciprofloxacin and sulfadimethoxine (measured in water samples), tetracycline and sulfadimethoxine (measured in sediment samples) and ARG abundance measured in both water and sediment (*bla*_SHV_, *sul1*, and *tet*(A)) did not show significant spatial autocorrelation. Results for all Moran’s I analyses are shown in Supplementary Tables S7–S10. For tetracycline concentrations in water, the Anselin’s Local Moran’s I test^20^ showed one significant high–high cluster and one significant low–low cluster, identifying locations that were closer together in distance and had similar tetracycline concentrations (Fig. 6).

**Figure 7:**
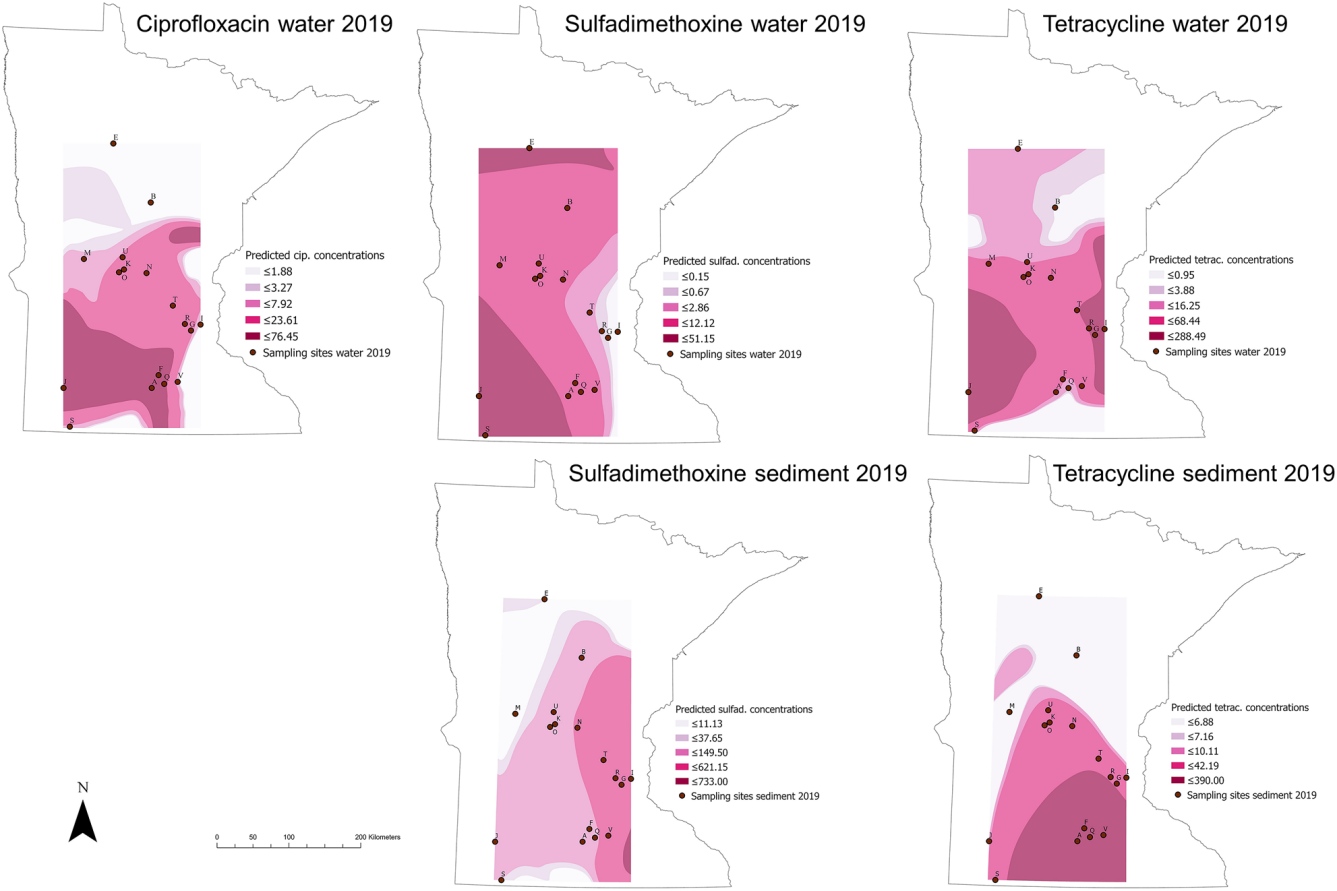
Contour maps resulting from the kernel interpolation for predicted antimicrobial concentrations in water and sediment samples in 2019 sampling. Sulfad.: sulfadimethoxine; cip: ciprofloxacin; tetrac.: tetracycline. Letters correspond to each one of the sampling sites (concentration at each site can be found in Supplementary Table S11).

**Figure 8:**
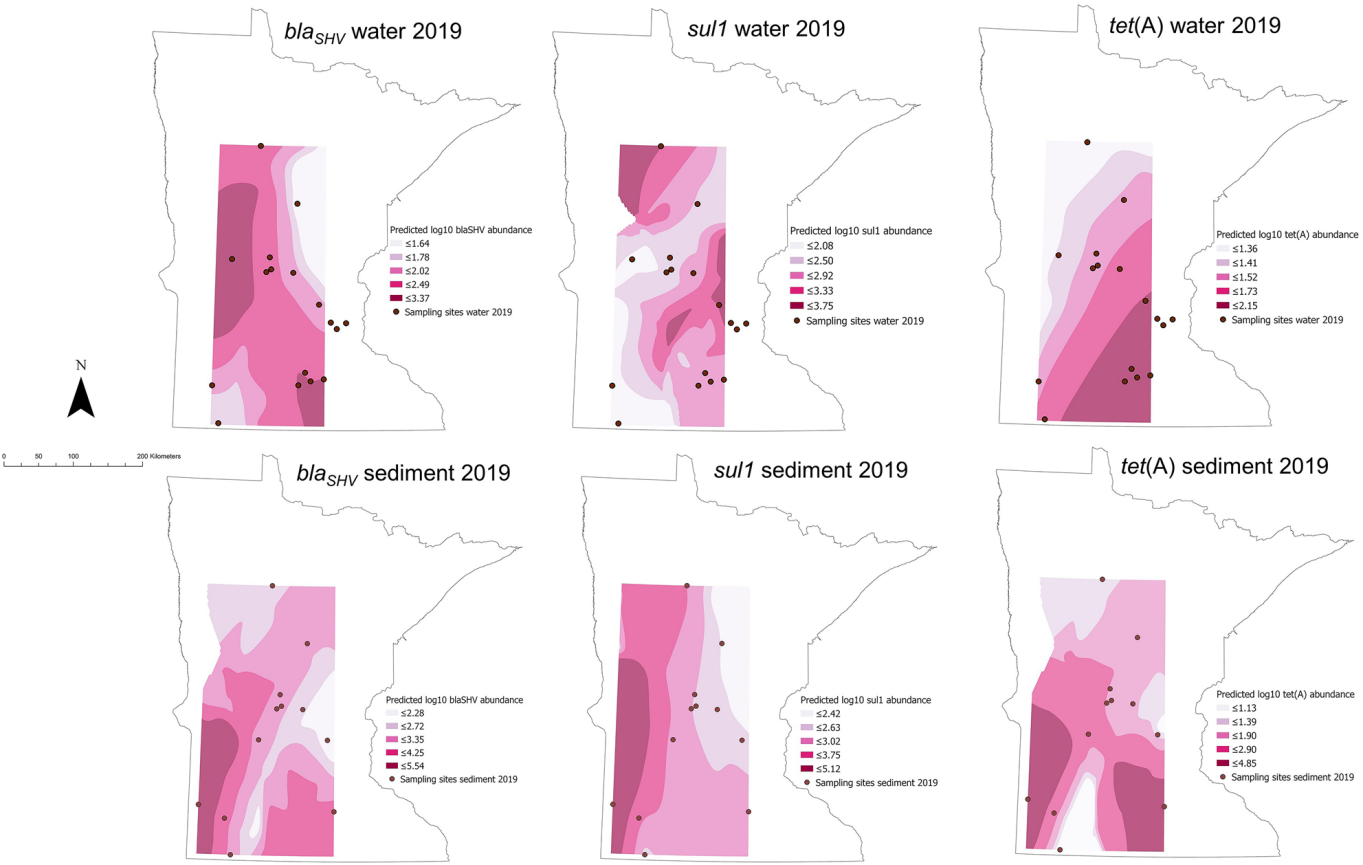
Contour maps resulting from the kernel interpolation for predicted antimicrobial resistance gene abundance in water and sediment samples in 2019 sampling.

Contour maps of predicted antimicrobial concentrations and ARG abundance across Minnesota, generated using the kernel interpolation method, are shown in Figs. 7 and 8 for each antimicrobial and ARG in water and sediment samples. Areas of the state without sampling locations appear empty in the maps, and the darker color is associated with higher predicted antimicrobial concentrations and ARG abundance. Kernel interpolation results provided an approximation of the range of antimicrobial concentrations and ARG abundance likely to be detected at unsampled locations. Interpolation was not limited to waterbody sites but provided predictions for areas surrounding the sampling sites within a specific bandwidth, which included areas of land. For antimicrobial concentrations, the highest predicted values for ciprofloxacin in water were in one area expanding from the south-west towards the south-central area of the state, and in a smaller area towards the north-eastern side. Sulfadimethoxine predicted values were highest in the northern areas and in the south-west in water samples, while in sediment, the highest predicted values were in a small area in the south-east. The highest predicted values for tetracycline in water samples were in the south-west towards the center and the central area expanding towards the northern and southern areas, while in sediment samples, the highest predicted values were in the south-east/central regions. Antimicrobial concentrations at each sampling site are found in Supplementary Table S11. For ARG abundance, the areas with highest predicted values for *bla*_SHV_ in water were in the central-western areas and south-central areas, while in sediment it was in the south-west; for *sul1* abundance in water, the highest predicted values were north-west and two areas in the central region, while in sediment it was central and south-west. Finally, for *tet*(A), the highest predicted abundance in water and sediment was in the south-east, and in the south-western area as well for sediment samples only (Figs. 7, 8).

### Microscale analysis

Associations between environmental and spatial factors and the antimicrobial concentrations in water samples (ciprofloxacin, tetracycline, and sulfadimethoxine) and sediment samples (tetracycline, and sulfadimethoxine) were assessed for 2019 samples (n = 19 water, n = 17 sediment). A moderate positive correlation was identified between water pH and sulfadimethoxine concentrations (*r* = 0.51, *p* = 0.04) in 2019 sediment samples (Supplementary Tables SS12–13).

There were moderate negative correlations between water temperature and *sul1* (*r* = − 0.43, *p* = 0.04), *sul2* (*r* = − 0.43, *p* = 0.04), and *tet*(A) (*r* = − 0.44, *p* = 0.03) abundance in 2019 water samples. There were also moderate negative correlations between water temperature and *intI3* (*r* = − 0.57, *p* = 0.01), *mexB* (*r* = − 0.54, *p* = 0.02), *strB* (*r* = − 0.57, *p* = 0.01), *sul3* (*r* = − 0.52, *p* = 0.03), and *tet*(A) (*r* = − 0.61, *p* = 0.007) abundance in 2019 sediment samples (Supplementary Tables S14–S15). The correlation analyses between ARG abundance and antimicrobial concentrations for 2019 in each sample type separately revealed moderate positive correlations between ciprofloxacin and *mexB* (*r* = 0.56, *p* = 0.004) in 2019 water samples, and between tetracycline and *tet*(A) (*r* = 0.43, *p* = 0.04) and sulfadimethoxine and *tet*(W) *(r* = 0.48, *p* = 0.02) for 2019 sediment samples (Supplementary Tables S16–S17).

There was no statistically significant effect of wastewater treatment discharge on antimicrobial concentrations in water or sediment samples in 2019 after adjusting for waterbody as a random effect in the Linear Mixed Models (LMMs). Antimicrobial concentrations, however, were highest at wastewater discharge sites compared to upstream and downstream sites at the same location. Upstream sites had higher concentrations of tetracycline in both water and sediment, and of sulfadimethoxine in water samples compared to downstream sites (Supplementary Tables S18–S19). In the case of ARG abundance, *sul1* (*p* = 0.0009) and *sul2* (*p* = 0.007) abundance in water samples, and *bla*_SHV_ (*p* = 0.0001), *intI1* (*p* = 0.02), *mexB* (*p* = 2.2 × 10^–16^), and *sul2* (*p* = 0.03) abundance in sediment samples were significantly higher at the discharge site (WT) compared to upstream (U) and downstream (D) sites. The estimated marginal mean (EMM) plots are shown in Supplementary Figures S5–S10.

ARG abundance did not yield any significant differences between the two time sampling events (T1: end of June–early July 2018, and T2: end of July to mid-August 2018) when adjusting for the random effect of waterbody site for water or sediment samples during 2018, except for *bla*_SHV_ (*p* = 1.84 × 10^–5^) and *intI1* (*p* = 2.48 × 10^–5^) in water, where there was a higher abundance at T2. The difference in gene abundance between T1 and T2 was expressed as the difference in EMM, based on the LMMs. The EMM measures represent gene abundance at each site adjusted for waterbody site and they are plotted in Supplementary Figures S11–S12.

## Discussion

Environmental monitoring is needed to advance our understanding of AMR persistence and dissemination. In this paper we describe an approach guided by spatial mapping to quantify and predict antimicrobials and ARG at two spatial scales (macro and micro) considering inputs from different sources and including two different sampling matrices (water and sediment) from waterbodies in Minnesota.

Two different approaches were used for field sampling: convenience (in 2018) and spatially-guided (in 2019). Convenience sampling involves a subjective selection of field sites based on the “ease of obtaining a sample”^21^. It has the advantages of selecting sites with easier access, more availability, and it is a useful approach when there are limited resources. However, it is more likely to introduce biases into the results, which tend to be mostly descriptive^22^. Spatially-guided sampling involves underlying knowledge about the population of interest. In our study, spatial mapping using Geographic Information Systems (GIS) was used to determine the spatial distribution of human and livestock densities, wastewater treatment plant and other point source locations, as well as to provide information about waterbody locations throughout the state. This diverse information informed the second field sampling season (in 2019), as had been suggested in an exploratory study conducted in Ireland^14^.

Spatial analysis findings were mostly descriptive given the relatively small sample size throughout the entire state, which did not allow for a more sophisticated interpolation method such as kriging. Instead, we used kernel interpolation, which is an indicated method for small datasets^23^. Kernel interpolation results provided an approximation of the range of antimicrobial concentrations and ARG abundance likely to be detected at unsampled sites. Interpolation was not limited to waterbody sites but provided predictions for areas surrounding the sampling sites within a specific bandwidth. Spatial analyses were also used to identify clusters of antimicrobial concentrations (e.g., tetracycline), meaning locations that were closer together in distance and had similar tetracycline concentrations. Further, by mapping the sampling sites, we were able to determine the locations with the highest antimicrobial concentrations and ARG abundance.

The two spatial scales provided different information. The macroscale analysis was useful to identify large areas throughout the state with higher human and/or animal density that could influence antimicrobial concentrations and ARG abundance, but it involved higher uncertainty and less precision. Antimicrobials with highest concentrations included oxytetracycline, norfloxacin, clarithromycin, and erythromycin. The first three antimicrobials were detected in higher concentrations in lakes near human populated areas. Finding higher concentrations of oxytetracycline in these areas could be explained by its use in companion animals (dogs and cats), because this antimicrobial is not used in human medicine^24–26^. Norfloxacin and clarithromycin belong to the quinolone and macrolide antibiotic classes, respectively, which are among the most prescribed antimicrobials in Minnesota outpatient settings^27^. Similar findings have been reported elsewhere. Norfloxacin is one of the most frequent quinolones detected in lakes around the world^7^. Clarithromycin has been detected in human waste in a river in South Korea^28^ and was widely detected in urban lakes in Vietnam^29^. Most of the research to date on antimicrobial concentrations in surface water has been conducted in Asia^7^. However, at the local level, the Minnesota Pollution Control Agency (MPCA) has conducted state-wide surveys during the past decade on emerging contaminants in the Minnesota environment. None of the three antimicrobials mentioned above (oxytetracycline, norfloxacin, and clarithromycin) were detected in quantifiable concentrations in a study focused on lakes and rivers^30^, and only clarithromycin was detected in a Minnesota site (Cedar River) in a study focused on rivers and streams^31^.

In our sediment samples, erythromycin was the compound with the highest concentration in a lake located in a state park, with minimal direct human or animal influence. This macrolide has been reported in high concentrations in lake water and sediment samples globally^7^. Erythromycin is used in humans and some animal species, and is also naturally occurring^32^, making it challenging to determine its specific origin. It is important to note though, that when comparing findings across studies, it is essential to keep in mind differences in laboratory methodology that can lead to erroneous comparisons. While the highest antimicrobial concentrations were found near human populated areas, sites with the highest ARG abundance were more widespread across waterbodies in the state, found both in areas near higher livestock density and areas without much human or animal influence.

Even though the highest antimicrobial concentrations were found near human populated areas, there was no association between human density and antimicrobial concentrations. There was no association with neither of the environmental parameters (water temperature, water pH, conductivity) in the 2019 water samples. This could be a result of the low power of our study, the variable nature of water samples, or that antimicrobial concentrations might not be influenced by these parameters in the locations sampled. However, among sediment samples, there was a moderate correlation between water pH and sulfadimethoxine concentration. Sulfonamide persistence and transport (including sulfadimethoxine) are reported to be influenced by pH in soil, with decreased pH values leading to increases in sorption potential of sulfonamides in soil^33^.

The microscale analysis was useful to assess the influence of a specific point source on the measured outcomes (antimicrobial concentration and ARG abundance), but the information gained from this analysis was limited to the specific sites where samples were collected. There was no significant effect of wastewater on antimicrobial concentrations, but the concentrations were highest at the wastewater discharge site compared to upstream and downstream sites at the same location. This could indicate a relatively short spatial distance affected by the discharge. In the case of ARG, the abundance of several genes was significantly higher at the wastewater discharge point compared to upstream and downstream sites. Genes encoding resistance to sulfonamides have been reported to be more abundant than other ARG in studies of different types of wastewater treatment plants^34^ and to be common in aquatic systems^11^. These genes (*sul1* and *sul2*) are very mobile, and along with tetracycline resistance genes are the most studied ARG in lakes and rivers^7^. In our study, *sul1* and *bla*_SHV_ were the most abundant ARG across the waterbodies investigated throughout Minnesota. The gene *bla*_SHV_, which confers resistance to beta-lactam antimicrobials and thus it is of public health concern, has been increasingly reported in environmental studies around the world, mostly near or at wastewater treatment plants^18,35^. Other commonly detected genes in our study included *tet*(A), which is frequently found in wastewater environments (including in previous studies within Minnesota waterbodies^36^), fish farm ponds, and swine lagoons^11^. Each of the spatial scales (macro and micro) underscores different aspects of environmental AMR, thus informing different opportunities for management and mitigation. Spatial scale has been previously discussed in the context of AMR in the environment, highlighting how causal factors important in the emergence, dissemination, and persistence of AMR can have spatial relevance at different scales^4,37,38^.

Both sediment and water samples were collected because they provide different information about antimicrobial and ARG presence in waterbodies. Because sediment can retain antimicrobial compounds and ARG longer than water, sediment samples reflect the long-term history of antimicrobials and ARG in that location. In fact, higher concentration of antimicrobials in sediment can indicate accumulation with the potential for subsequent release into the water matrix^39,40^. Water samples reflect a more transient record of antimicrobial and ARG^32,39,40^. Laboratory quantification of antimicrobials in sediment proved more challenging than in water samples due to the more complicated matrix, leading to difficulties with chromatography and higher background noise in the detector. Even though we did not evaluate the relationship between water and sediment for the same sites in this study, past studies have shown that pharmaceuticals and pesticide concentrations in water and sediment are decoupled due to the differences in water and sediment residence time and transport^41^.

For ARG, *sul1, bla*_SHV_*, intI1,* and *mexB* had the highest abundance in both water and sediment samples, potentially reflecting the ubiquity of some ARG across environmental matrices. This could also reflect the ability of these genes to be transferred within aquatic environments^42^. There were specific genes, however, detected only in one of the two sampling matrices. Specifically*, bla*_OXA_, *impl3*, *intI3*, and *qacF* were only detected in water, while *ermB*, *mefE* and *tet*(W) were only detected in sediment. This type of finding has been previously associated with the unique environmental conditions in each environmental matrix and to the existence of specific microorganisms in each one of the matrices^43^. Even though *bla*_OXA_ was only found in water samples in 2018 at a low frequency across samples (21%), this gene is of importance for public health because it encodes for resistance to carbapenems, antimicrobials of last resort^44^.

One limitation to the interpretation of data collected in this study is that, in addition to lakes, samples were collected from rivers and creeks, because in some cases, these were the compartments up or downstream of a point source. Hydrologically, these waterbody types differ, including water flow dynamics, which may affect the retention times for antimicrobials and ARG. We were not able to compare the effect of waterbody type given the sample size limitations, but it is important to take this factor into account when assessing the results and planning future field studies. In addition, the spatial approach we used to make predictions at the macroscale was not as powerful as other statistical approaches such as kriging, which could have been conducted with a higher number of sampling sites. Further, the interpolation method we used did not discriminate between waterbodies and areas of land, which needs to be considered when interpreting the predictions.

Work is ongoing to improve these models through incorporation of additional sampling locations, and with an estimation of the spatial distribution of antimicrobial use for both animals and humans. Application of known prescribing and/or administration rates by drug class to mapped human and animal population densities will allow for inclusion of proportional antimicrobial inputs into the model. This approach will support spatial predictions of antimicrobial and ARG loads in the environment. In this study, we only used density of animals and humans as a proxy predictor of overall antimicrobial release, but this simplistic approach can be improved with antimicrobial-use data. Targeted field sampling and analysis efforts will also be essential to improve the resolution at the microscale. This detail is necessary to understand the influence of specific point sources of antimicrobials and ARG and to model potential mitigation strategies. Increased sampling on a smaller geographic scale will also allow for incorporation of time as a measure of impact. Assessment of temporal factors is critical to correlate environmental findings with fluctuations in antimicrobial prescribing and the natural environment throughout the year.

In conclusion, this study highlights the value of using spatial analyses, different spatial scales, and sampling matrices, to design an environmental monitoring approach to advance our understanding of AMR persistence and dissemination. It also outlines the multiple professional disciplines and technical approaches needed to develop a comprehensive monitoring program for AMR and antimicrobials in the natural environment. A multidisciplinary approach to AMR ensures that all sectors (e.g., human health, clinic-based veterinary medicine, animal agriculture, crop management) that use antimicrobials are included in both the delineation of the problem and the identification of feasible solutions to mitigate release of antimicrobials and ARG into the natural environment. A collaborative, multisectoral approach at all levels-science, policy, and public engagement, is critical to protect human, animal, and ecosystem health.

## Methods

### Study area, study design, and sample collection

Water and sediment samples were collected from waterbodies (lakes, rivers, streams, creeks) throughout the state of Minnesota in the summers (June through August) of 2018 and 2019 to quantify antimicrobials and ARG. Summer was selected for sample collection as it is the only time of the year when this is feasible in Minnesota. Sample sites were selected by convenience in 2018. In 2019, spatial data for point sources such as wastewater treatment plants and animal agriculture locations, as well as other landscape features, were used to inform the sampling design. Sites in 2019 were selected at both a macro and micro scale. For the macroscale, study areas were identified throughout the state based on human and/or livestock densities. Within each of these study areas, points were identified where there was direct discharge of a wastewater treatment plant into a waterbody. The microscale sampling locations were then selected upstream, at the wastewater discharge, and downstream from the effluent with a maximum distance of 2 km spanning these microscale sites (Fig. 4). Additional details regarding sample collection procedures and locations are found in the Supplementary Information (Supplementary Methods and Supplementary Table S20).

### Laboratory work

#### Antimicrobials

Chemical sources and purities relevant to antimicrobial extraction and measurement are reported in the Supplementary Information. Water samples were concentrated using solid phase extraction (SPE) using a method adapted from Kerrigan et al.(2018) and Meyer et al. (2007)^32,45^. Freeze dried sediment samples were solvent extracted with assistance of ultrasound and then cleaned up using SPE. Both water and sediment extracts were analyzed using liquid chromatography tandem mass spectrometry. Details of the procedures, including calibration and determination of limits of detection and quantification are presented in the Supplementary Information (Supplementary Methods and Supplementary Tables S21–S23).

#### Antimicrobial resistance genes

Filters obtained with the REXEED 25S ultrafiltration system in the field were back-flushed and concentrated as previously described^46^. The final concentrated solution (average volume = 9.6 ± 5.3 mL) was stored at − 20 °C until DNA extraction. To extract DNA, a small aliquot of the concentrated solution (0.3 mL) or sediment (average sample volume = 504 ± 29.7 mg) was mixed with 0.7 mL of lysis buffer (5% sodium dodecyl sulfate, 120 mM sodium phosphate buffer, pH 8). Mixed samples then underwent three freeze/thaw cycles followed by 90 min of incubation at 70 °C to lyse cells and release metagenomic DNA. DNA was then purified using the FastDNA™ kit (MP Biomedicals, Santa Ana, California, USA) as per manufacturer’s instructions.

A total of 20 ARG representing different molecular mechanisms of resistance and different antimicrobial classes were selected for this study. In addition, the 16S ribosomal RNA (rRNA) gene and integrase genes (*intI1*, *intI2*, and *intI3*) were included. Microfluidic qPCR (MF-qPCR) was performed as described previously^47^. Prior to MF-qPCR, every target gene except for 16S rRNA was amplified using the specific target amplification (STA)^47^. Primers and standard gBlocks® sequences used for this study are presented in Supplementary Table S24. The limit of quantification (LOQ) for the assay was 1000 copies/µL of DNA for the target gene *mexB*, and 10 copies/µL of DNA for the rest of the target genes. Non-detects, defined as reactions that failed to produce a minimum amount of signal^48^, were replaced with ½ LOQ for those ARG that had non-detects in < 80% of the samples^18,19^. Those target genes that had ≥ 80% of non-detects across samples were removed from further analyses.

### Data analyses

#### Data sources

Livestock density at the state level was obtained from the Minnesota Pollution Control Agency (MPCA)^49^. These datasets included the geocoded location of the farms as well as the animal species and permitted number of total animal units on each farm^50^. Human population density at the census track level for Minnesota was obtained from the National Historical Geographic Information System (NHGIS)^51^, and wastewater treatment facility locations were obtained from the MPCA^52^. Human density, livestock density (broken out into swine, beef cattle, dairy, turkey, and chicken farms), wastewater treatment plant, ethanol plant, and hospital locations were mapped using ArcGIS Pro version 2.6.0. (ESRI®). Livestock density and human density data were extracted from a 5 km buffer around each sampling point. A small buffer size such as this was chosen to avoid the influence of other potential factors and sources on the outcome (antimicrobial concentrations and ARG abundance).

Descriptive statistics were conducted for both antimicrobials and ARG for each sample type (water and sediment) and year (2018 and 2019) separately. Antimicrobial concentrations (log_10_ transformed prior to analyses to meet normality assumptions) and ARG abundance (expressed as absolute abundance as log_10_ gene copies/L water or as log_10_ gene copies/g sediment) from the 2019 data were evaluated individually for their association with environmental parameters (water temperature, pH, and conductivity) and with spatial data (human density, livestock density, and wastewater discharge) using Pearson correlation with the cor.test function from the stats package in R^53^. Alpha was set at 5% for statistical significance. From all antimicrobials analyzed in the laboratory, ciprofloxacin, tetracycline, and sulfadimethoxine were evaluated for water sample data analyses, while tetracycline and sulfadimethoxine were evaluated for sediment sample analyses. These antimicrobials were chosen as they represent different antimicrobial classes, have diverse chemistry, and have different use patterns (the fluoroquinolone class is often used in humans, and, at times, in companion animals and some livestock; tetracycline is broadly used across all animal species, including livestock; and sulfadimethoxine is used in veterinary medicine^24,54–58^). Missing environmental parameters at sampling sites (water temperature, pH, conductivity) were imputed using the mean value of each parameter^59^. ARG abundance for 2019 was further evaluated for potential correlations with antimicrobial concentrations for each sample type. For these analyses, the function rcorr from the Hmisc package^60^ and the package corrplot^61^ were used, and an alpha was set at 5% for statistical significance.

For those waterbodies in 2019 with direct wastewater effluent discharge, the effect of the effluent on antimicrobial concentrations as well as on ARG abundance in water and sediment samples was evaluated by fitting linear mixed regression models (LMMs) to the log_10_ transformed data using the lme4^62^ and lmerTest packages^63^ in R with the function lmer. In these models, waterbody was included as random effect, given the repeated sampling from the same locations in both 2018 and 2019, and site (upstream or U, downstream or D, wastewater discharge or WT) as a fixed effect, with upstream (U) as the reference level. Statistical significance was defined with an alpha level of 5%, and Satterthwaite's approximation was used to obtain the p values for the F test for each model^64^. Model assumptions were checked through the inspection of residual plots^65^. ARG abundance was also compared between the two-time sampling points for 2018 fitting LMMs as described previously. In these models, waterbody was included as a random effect, and time (T1: end of June–early July 2018, T2: end of July to mid-August 2018) as a fixed effect, with T1 as the reference level.

To assess spatial dependency, the Global Moran’s I global clustering test was performed for each of the three antimicrobials stated above (ciprofloxacin, tetracycline, and sulfadimethoxine) and for the three ARG with the highest abundance. For the Moran’s I global spatial measure, the null hypothesis tested was that there was no spatial autocorrelation across the study area^66^. An average fixed distance band of 30,765 m for water sample sites, and of 35,081 m for sediment sample sites, and Euclidean distance were used for Moran’s I. The distance band was estimated using the tool ‘Calculate Distance Band from Neighbor Count’ from the Spatial Statistics toolbox of ArcGIS Pro (ESRI®). This tool determines the average distance between sampling locations (each location needs to have a minimum of one neighbor for Moran’s I). If there was global clustering, the Anselin’s Local Moran’s I (LISA) test was used to indicate the physical location of the clustering, given Global Moran’s I does not provide that level of detail^20^. For LISA, the same fixed distance band was used. The kernel interpolation (a.k.a. kernel smoothing) tool was used to visualize the antimicrobial concentration outcomes and ARG abundance from 2019 and to predict the antimicrobial concentrations and ARG abundance respectively beyond the sampling sites. Kernel interpolation was conducted using default parameters which can be found in Supplementary Tables S25–S28. All geospatial analyses and mapping were conducted using ArcGIS Pro version 2.6.0. (ESRI®) and statistical analyses were conducted using R version 3.6.0^53^.

## Supplementary Information

Supplementary Information.

## Data Availability

All measurements of antimicrobial concentrations in water and sediment are available in the Data Repository for the University of Minnesota at 10.13020/xcbx-t731. The other data used to support the findings of this study are available from the corresponding author upon request.
