# Supplementary material for: Quantifying and predicting antimicrobials and antimicrobial resistance genes in waterbodies through a holistic approach: a study in Minnesota, United States
Source: Sci Rep. 2021 Sep 21;11:18747. doi: 10.1038/s41598-021-98300-5 (PMC8455696; doi:10.1038/s41598-021-98300-5)
Supplement: Supplementary file 1 — Supplementary Information. [file 41598_2021_98300_MOESM1_ESM.docx]

**Supplementary Information**

Quantifying and predicting antimicrobials and antimicrobial resistance genes in waterbodies through a holistic approach: a study in Minnesota, United States

Irene Bueno^1*^, Amanda Beaudoin^1,2^, William A. Arnold^3,4^, Taegyu Kim^3^, Lara E. Frankson^4^, Timothy M. LaPara^3^, Kaushi Kanankege^5^, Kristine H. Wammer^6^, Randall S. Singer^1^

1. Department of Veterinary and Biomedical Sciences, College of Veterinary Medicine, University of Minnesota. 1971 Commonwealth Ave. St. Paul, MN, 55108. United States.

2. Minnesota Department of Health. P.O. Box 64975, St. Paul, MN 55164-0975. United States.

3. Department of Civil, Environmental, and Geo-Engineering, University of Minnesota. 500 Pillsbury Dr. SE, Minneapolis, MN 55455. United States.

4. Water Resources Science Program. University of Minnesota, 1985 Buford Ave., St. Paul, MN 55108. United States.

5. Center for Animal Health and Food Safety, College of Veterinary Medicine, University of Minnesota, 1354 Eckles Ave., St. Paul, MN, 55108. United States.

6. College of Arts & Sciences, University of St. Thomas. 2115 Summit Ave. St. Paul, MN, 55105. United States.

*Corresponding author: Irene Bueno. e-mail: bueno004@umn.edu

Table S1. Antimicrobial resistance genes (ARG) and integron genes that were removed from the analyses because they had non-detects in ≥80% of the water samples for 2018 and 2019. Numbers indicate non-detect as a percentage %, and as the number of samples (n).

| ARG | 2018 | 2019 |
| --- | --- | --- |
| *ampC* | 87% (33) | 88% (21) |
| *ereB* | 97% (37) | 100% (24) |
| *ermB* | 87% (33) | 88% (21) |
| *ermF* | 92% (35) | 96% (23) |
| *mefE* | 84% (32) | 96% (23) |
| *qnrA* | 98% (30) | 100% (24) |
| *tet*(M) | 92% (35) | 100% (24) |
| *tet*(W) | 82% (31) | 96% (23) |
| *tet*(X) | 87% (33) | 96% (23) |
| *vanA* | 92% (35) | 100% (24) |
| *intI2* | 89% (34) | 96% (23) |

Table S2. Antimicrobial resistance genes (ARG) and integron genes that were removed from the analyses because they had non-detects in ≥80% of the sediment samples for 2018 and 2019. Numbers indicate non-detect as a percentage %, and as the number of samples (n).

| ARG | 2018 | 2019 |
| --- | --- | --- |
| *ampC* | 100% (33) | 100% (30) |
| *bla*_OXA_ | 91% (30) | 90% (27) |
| *ereB* | 97% (32) | 90% (27) |
| *ermF* | 97% (32) | 90% (27) |
| *IMP-13* | 97% (32) | 97% (29) |
| *mefE* | 88% (29) | - |
| *qacF* | 100% (33) | 93% (28) |
| *qnrA* | 100% (33) | 97% (29) |
| *sul2* | 88% (29) | - |
| *sul3* | 82% (27) | - |
| *tet*(M) | 100% (33) | 93% (28) |
| *tet(*W) | 94% (31) | - |
| *tet*(X) | 100% (33) | 93% (28) |
| *vanA* | 97% (32) | 100% (30) |
| *intI2* | 100% (33) | 97% (29) |
| *intI3* | 82% (27) | - |

Table S3. Summary statistics (arithmetic mean of log values±standard error, standard deviation (SD), median, and range: minimum (min) and maximum (max)) for antimicrobial resistance gene (ARG) abundance (log_10_ gene copies/L water) analyzed in water samples across sites in 2018 (n=22).

| ARG | Mean± SE | SD | Median | Range: min, max |
| --- | --- | --- | --- | --- |
| *bla*_OXA_ | 1.5±0.0 | 0.2 | 1.5 | 1.1, 2.0 |
| *bla*_SHV_ | 2.6±0.2 | 0.8 | 2.6 | 1.4, 3.7 |
| *IMP-13* | 1.6±0.1 | 0.3 | 1.5 | 1.2, 2.3 |
| *intI1* | 2.9±0.2 | 1.1 | 2.8 | 1.4, 5.3 |
| *intI3* | 1.6±0.1 | 0.4 | 1.5 | 1.1, 3.2 |
| *mexB* | 2.4±0.1 | 0.5 | 2.5 | 1.2, 3.7 |
| *qacF* | 1.6±0.1 | 0.6 | 1.5 | 1.0, 3.9 |
| *strB* | 1.7±0.1 | 0.7 | 1.5 | 1.2, 4.0 |
| *sul1* | 2.5±0.3 | 1.2 | 2.2 | 1.3, 5.9 |
| *sul2* | 1.9±0.2 | 0.8 | 1.5 | 1.1, 4.3 |
| *sul3* | 1.5±0.0 | 0.1 | 1.5 | 1.2, 1.6 |
| *tet*(A) | 1.7±0.1 | 0.6 | 1.5 | 1.2, 3.5 |

Table S4. Summary statistics ((arithmetic mean of log values±standard error, standard deviation (SD), median, and range: minimum (min) and maximum (max)) for antimicrobial resistance gene (ARG) abundance (log_10_ gene copies/L water) analyzed in water samples across sites in 2019 (n=10).

| ARG | Mean± SE | SD | Median | Range: min, max |
| --- | --- | --- | --- | --- |
| *bla*_SHV_ | 2.1±0.2 | 0.6 | 1.9 | 1.4, 3.4 |
| *IMP-13* | 1.5±0.0 | 0.1 | 1.5 | 1.3, 1.7 |
| *intI1* | 2.0±0.2 | 0.8 | 1.8 | 1.3, 3.5 |
| *intI3* | 1.5±0.0 | 0.1 | 1.5 | 1.3, 1.7 |
| *mexB* | 2.5±0.1 | 0.2 | 2.5 | 2.0, 2.8 |
| *strB* | 1.5±0.1 | 0.2 | 1.5 | 1.4, 1.9 |
| *sul1* | 2.6±0.2 | 0.6 | 2.5 | 1.7, 3.7 |
| *sul2* | 1.7±0.1 | 0.3 | 1.7 | 1.3, 2.1 |
| *tet*(A) | 1.6±0.1 | 0.2 | 1.5 | 1.3, 2.1 |

Table S5. Summary statistics (arithmetic mean of log values ±standard error, standard deviation (SD), median, and range: minimum (min) and maximum (max)) for antimicrobial resistance gene (ARG) abundance (log_10_ gene copies/g sediment) analyzed in sediment samples across sites in 2018 (n=20).

| ARG | Mean± SE | SD | Median | Range: min, max |
| --- | --- | --- | --- | --- |
| *bla*_SHV_ | 2.8±0.2 | 1.0 | 2.7 | 1.6, 4.3 |
| *ermB* | 1.7±0.1 | 0.7 | 1.6 | 0.2, 3.4 |
| *intI1* | 2.5±0.2 | 0.9 | 2.5 | 1.5, 4.5 |
| *mexB* | 2.6±0.1 | 0.7 | 2.7 | 0.9, 3.7 |
| *strB* | 1.4±0.1 | 0.5 | 1.6 | 0.3, 1.7 |
| *sul1* | 2.1±0.2 | 0.8 | 2.1 | 0.4, 3.5 |
| *tet*(A) | 2.1±0.2 | 0.7 | 2.1 | 0.6, 3.5 |

Table S6. Summary statistics (arithmetic mean of log values ±standard error, standard deviation (SD), median, and range: minimum (min) and maximum (max)) for antimicrobial resistance gene (ARG) abundance (log_10_ gene copies/g sediment) analyzed in sediment samples across sites in 2019 (n=12).

| ARG | Mean± SE | SD | Median | Range: min, max |
| --- | --- | --- | --- | --- |
| *bla*_SHV_ | 3.1±0.3 | 0.9 | 3.1 | 1.7, 5.5 |
| *ermB* | 2.0±0.3 | 0.9 | 1.7 | 1.1, 4.4 |
| *intI1* | 2.3±0.4 | 1.4 | 2.1 | 0.4, 5.4 |
| *intl3* | 1.8±0.3 | 1.0 | 1.7 | 0.0, 3.9 |
| *mefE* | 1.6±0.2 | 0.8 | 1.4 | 0.6, 3.8 |
| *mexB* | 2.7±0.3 | 1.2 | 2.5 | 1.2, 4.6 |
| *strB* | 1.7±0.2 | 0.8 | 1.6 | 0.6, 3.6 |
| *sul1* | 2.8±0.2 | 0.8 | 2.6 | 2.0, 5.1 |
| *sul2* | 1.8±0.3 | 0.9 | 1.6 | 0.9, 4.3 |
| *sul3* | 1.8±0.2 | 0.8 | 1.7 | 0.5, 3.5 |
| *tet*(A) | 2.1±0.3 | 1.2 | 1.8 | 1.0, 4.9 |
| *tet*(W) | 1.0±0.2 | 0.6 | 1.2 | 0.0, 1.7 |

Table S7. Global Moran’s I results for the three antimicrobials (ciprofloxacin, tetracycline, and sulfadimethoxine) for 2019 water samples averaged across the same waterbody.

| Antibiotic | z-score | p-value | Moran’s Index |
| --- | --- | --- | --- |
| Ciprofloxacin | 1.62 | 0.10 | 0.35 |
| Tetracycline | 2.30 | 0.02 | 0.50 |
| Sulfadimethoxine | 0.88 | 0.38 | 0.14 |

Table S8. Global Moran’s I results for the two antimicrobials (tetracycline, and sulfadimethoxine) for 2019 sediment samples averaged across the same waterbody.

| Antibiotic | z-score | p-value | Moran’s Index |
| --- | --- | --- | --- |
| Tetracycline | -0.09 | 0.93 | -0.07 |
| Sulfadimethoxine | 1.53 | 0.13 | 0.32 |

Table S9. Global Moran’s I results for the three antimicrobial resistance genes (ARG) (*bla*_SHV,_ *sul1,* and *tet(*A)) for 2019 water samples averaged across the same waterbody.

| ARG | z-score | p-value | Moran’s Index |
| --- | --- | --- | --- |
| *bla*_SHV_ | -0.20 | 0.84 | -0.20 |
| *sul1* | 0.07 | 0.94 | -0.08 |
| *tet*(A) | -0.03 | 0.97 | -0.13 |

Table S10. Global Moran’s I results for the three antimicrobial resistance genes (ARG) (*bla*_SHV,_ *sul1,* and *tet(*A)) for 2019 sediment samples averaged across the same waterbody.

| ARG | z-score | p-value | Moran’s Index |
| --- | --- | --- | --- |
| *bla*_SHV_ | 0.62 | 0.53 | 0.11 |
| *sul1* | -1.47 | 0.14 | -0.50 |
| *tet*(A) | -0.16 | 0.87 | -0.03 |

Table S11. Antimicrobial concentrations in water (ng/L) and sediment (ng/g) in 2019 for ciprofloxacin, sulfadimethoxine, and tetracycline at sampling sites.

| Site | Name | Ciproflox. water | Sulfadime. water | Tetra. water | Sulfadime. sediment | Tetra. sediment |
| --- | --- | --- | --- | --- | --- | --- |
| A | Blue Earth River | 40.1 | 0.4 | 0.4 | 7.9 | 5.3 |
| B | Crosslake | 1.5 | 0.1 | 0.4 | 6.4 | 4.9 |
| C | Florida Slough Lake | 12.4 | 4.5 | 61.0 |  |  |
| D | Lake Andrew | 12.4 | 2.9 | 130.8 |  |  |
| E | Lake Bemidji | 1.5 | 20.4 | 7.9 | 13.4 | 3.9 |
| F | Lake Emily | 40.1 | 0.4 | 0.4 | 11.5 | 52.0 |
| G | Lake Harriet | 3.5 | 1.4 | 145.0 | 252.0 | 40.0 |
| H | Lake Ida | 2.9 | 1.2 | 6.8 |  |  |
| I | Lake Owasso | 3.5 | 2.7 | 121.1 | 652.0 | 7.5 |
| J | Lake Shetek | 58.6 | 16.4 | 288.5 | 162.4 | 12.9 |
| K | Lake St. Anna |  |  |  | 11.5 | 7.1 |
| L | Lake Wakanda | 2.2 | 28.0 | 32.5 |  |  |
| M | Lake Winona | 2.9 | 0.3 | 18.8 | 7.4 | 4.2 |
| N | Little Rock Lake | 1.7 | 4.7 | 0.5 | 345.5 | 8.3 |
| O | Long Lake |  |  |  | 90.5 | 33.7 |
| P | Long Prairie River | 2.9 | 0.7 | 1.0 |  |  |
| Q | Madison Lake | 40.1 | 0.4 | 0.4 | 4.8 | 390 |
| R | Medicine Lake | 3.5 | 1.4 | 66.2 | 733.0 | 7.5 |
| S | Ocheda Lake | 2.3 | 51.1 | 0.3 | 7.7 | 14.7 |
| T | Otsego Creek | 76.5 | 0.0 | 3.5 | 7.9 | 5.3 |
| U | Pepin Lake |  |  |  | 11.5 | 7.1 |
| V | Sakatah Lake | 2.5 | 5.1 | 2.1 | 398.3 | 21.8 |

Table S12. Correlation results (*r* coefficients) for environmental and spatial predictors with antimicrobial concentrations in water samples collected in 2019 (n=19): ciprofloxacin, tetracycline, and sulfadimethoxine (p-values in parentheses).

|  | Water temperature | Water pH | Conductivity | Animal Units | Human density | Ciprofloxacin | Tetracycline | Sulfadimethoxine |
| --- | --- | --- | --- | --- | --- | --- | --- | --- |
| Water temperature |  | 0.03  (0.91) | -0.42  (0.07) | -0.06 (0.80) | -0.03 (0.91) | 0.22  (0.36) | 0.20  (0.41) | 0.36  (0.13) |
| Water pH | 0.03  (0.91) |  | -0.06  (0.79) | -0.34 (0.14) | 0.47 (0.04) | -0.09  (0.71) | 0.15  (0.54) | -0.36  (0.13) |
| Conductivity | -0.42  (0.07) | -0.06 (0.79) |  | 0.38 (0.11) | 0.32 (0.17) | 0.008  (0.97) | 0.28  (0.23) | -0.10  (0.69) |
| Animal Units | -0.06  (0.80) | -0.34 (0.14) | 0.38  (0.11) |  | -0.47 (0.04) | 0.24  (0.32) | 0.10  (0.69) | 0.30  (0.19) |
| Human density | -0.03  (0.91) | 0.47 (0.04) | 0.32  (0.17) | -0.47 (0.04) |  | -0.002  (0.99) | 0.29  (0.22) | -0.31  (0.18) |
| Ciprofloxacin | 0.22  (0.36) | -0.09 (0.71) | 0.008  (0.97) | 0.24 (0.32) | -0.002 (0.99) |  | -0.02  (0.92) | -0.29  (0.21) |
| Tetracycline | 0.20  (0.41) | 0.15 (0.54) | 0.28  (0.23) | 0.10 (0.69) | 0.29 (0.22) | -0.02  (0.92) |  | 0.18  (0.45) |
| Sulfadimethoxine | 0.36  (0.13) | -0.36 (0.13) | -0.10  (0.69) | 0.30 (0.19) | -0.31 (0.18) | -0.29  (0.21) | 0.18  (0.45) |  |

Table S13. Correlation results (*r* coefficients) for environmental and spatial predictors with antimicrobial concentrations in sediment samples collected in 2019 (n=17): ciprofloxacin, tetracycline, and sulfadimethoxine (p-values in parentheses).

|  | Water  temperature | Water pH | Conductivity | Animal  Units | Human  density | Tetracycline | Sulfadimethoxine |
| --- | --- | --- | --- | --- | --- | --- | --- |
| Water  temperature |  | -0.02  (0.95) | -0.65  (0.005) | 0.25  (0.34) | -0.39  (0.13) | 0.28  (0.28) | 0.18  (0.49) |
| Water pH | -0.02  (0.95) |  | -0.19  (0.46) | -0.15  (0.58) | 0.14  (0.60) | 0.26  (0.31) | 0.51  (0.04) |
| Conductivity | -0.65  (0.005) | -0.19  (0.46) |  | 0.17  (0.51) | 0.31  (0.23) | -0.20  (0.45) | -0.10  (0.69) |
| Animal Units | 0.25  (0.34) | -0.15  (0.58) | 0.17  (0.51) |  | -0.47  (0.06) | 0.33  (0.20) | -0.38  (0.14) |
| Human density | -0.39  (0.13) | 0.14  (0.60) | 0.31  (0.23) | -0.47  (0.06) |  | -0.12  (0.65) | 0.33  (0.19) |
| Tetracycline | 0.28  (0.28) | 0.26  (0.31) | -0.20  (0.45) | 0.33  (0.20) | -0.12  (0.65) |  | -0.007  (0.98) |
| Sulfadimethoxine | 0.18  (0.49) | 0.51  (0.04) | -0.10  (0.69) | -0.38  (0.14) | 0.33  (0.19) | -0.007  (0.98) |  |

Table S14. Correlation results (*r* coefficients) for environmental and spatial predictors with antimicrobial resistance gene abundance (log_10_ copies/L) in water samples (n=10) collected in 2019 (p-values in parentheses).

|  | *bla*_SHV_ | *IMP-13* | *intI1* | *intI3* | *mexB* | *strB* | *sul1* | *sul2* | tet*(A)* | Water pH | Water temperature | Conductivity | Animal Units | Human density |
| --- | --- | --- | --- | --- | --- | --- | --- | --- | --- | --- | --- | --- | --- | --- |
| *bla*_SHV_ |  | 0.23 (0.29) | 0.35 (0.1) | -0.16 (0.45) | 0.17 (0.43) | 0.24 (0.26) | 0.24 (0.25) | 0.4 (0.05) | 0.19 (0.34) | 0.07 (0.74) | -0.28  (0.19) | -0.04  (0.84) | -0.21 (0.32) | 0.14 (0.51) |
| *IMP-13* | 0.23 (0.29) |  | 0.44 (0.03) | 0.43 (0.03) | 0.56 (0.005) | 0 (0.99) | 0.05 (0.83) | -0.03 (0.88) | 0.52 (0.009) | 0.11 (0.62) | -0.23  (0.29) | -0.09  (0.68) | -0.19 (0.37) | -0.16 (0.47) |
| *intI1* | 0.35 (0.1) | 0.44 (0.03) |  | 0.3 (0.16) | 0 (0.99) | 0.26 (0.22) | 0.31 (0.14) | 0.2 (0.35) | 0.5 (0.01) | 0.14 (0.51) | -0.4  (0.05) | -0.17  (0.42) | -0.08 (0.72) | -0.08 (0.72) |
| *intI3* | -0.16 (0.45) | 0.43 (0.03) | 0.3 (0.16) |  | -0.03 (0.88) | 0.4 (0.55) | 0.24 (0.25) | 0.1 (0.66) | 0.21 (0.34) | -0.13 (0.56) | -0.04  (0.87) | -0.2  (0.36) | -0.12 (0.56) | -0.17 (0.43) |
| *mexB* | 0.17 (0.43) | 0.56 (0.005) | 0 (0.99) | -0.03 (0.88) |  | -0.33 (0.11) | -0.41 (0.05) | -0.54 (0.006) | 0.27 (0.20) | 0.03 (0.89) | 0.09  (0.67) | 0.2  (0.35) | -0.06 (0.77) | -0.33 (0.11) |
| *strB* | 0.24 (0.26) | 0 (0.99) | 0.26 (0.22) | 0.4 (0.55) | -0.33 (0.11) |  | 0.2 (0.36) | 0.52 (0.009) | 0.35 (0.09) | 0.07 (0.73) | -0.21  (0.33) | 0.06  (0.77) | -0.12 (0.57) | -0.18 (0.39) |
| *sul1* | 0.24 (0.25) | 0.05 (0.83) | 0.31 (0.14) | 0.24 (0.25) | -0.41 (0.05) | 0.2 (0.36) |  | 0.65 (0.0005) | 0.25 (0.23) | -0.3 (0.15) | -0.43  (0.04) | -0.06  (0.77) | -0.06 (0.77) | 0.4 (0.06) |
| *sul2* | 0.4 (0.05) | -0.03 (0.88) | 0.2 (0.35) | 0.1 (0.66) | -0.54 (0.006) | 0.52 (0.009) | 0.65 (0.0005) |  | 0.4 (0.05) | -0.03 (0.88) | -0.43  (0.04) | -0.01  (0.96) | -0.06 (0.77) | 0.37 (0.07) |
| *tet*(A) | 0.19 (0.34) | 0.52 (0.009) | 0.5 (0.01) | 0.21 (0.34) | 0.27 (0.20) | 0.35 (0.09) | 0.25 (0.23) | 0.4 (0.05) |  | -0.01 (0.95) | -0.44  (0.03) | 0.22  (0.31) | -0.02 (0.91) | -0.17 (0.43) |
| Water pH | 0.07 (0.74) | 0.11 (0.62) | 0.14 (0.51) | -0.13 (0.56) | 0.03 (0.89) | 0.07 (0.73) | -0.3 (0.15) | -0.03 (0.88) | -0.01 (0.95) |  | 0.06  (0.77) | -0.18  (0.4) | -0.2 (0.35) | 0.22 (0.28) |
| Water temperature | -0.28 (0.19) | -0.23 (0.29) | -0.4 (0.05) | -0.04 (0.87) | 0.09 (0.67) | -0.21 (0.33) | -0.43 (0.04) | -0.43 (0.04) | -0.44 (0.03) | 0.06 (0.77) |  | -0.07  (0.75) | 0.23 (0.27) | -0.16 (0.47) |
| Conductivity | -0.04 (0.84) | -0.09 (0.68) | -0.17 (0.42) | -0.2 (0.36) | 0.2 (0.35) | 0.06 (0.77) | -0.06 (0.77) | -0.01 (0.96) | 0.22 (0.31) | -0.18 (0.4) | -0.07  (0.75) |  | 0.58 (0.003) | -0.21 (0.33) |
| Animal Units | -0.21 (0.32) | -0.19  (0.37) | -0.08 (0.72) | -0.12 (0.56) | -0.06 (0.77) | -0.12 (0.57) | -0.06 (0.77) | -0.06 (0.77) | -0.02 (0.91) | -0.2 (0.35) | 0.23  (0.27) | 0.58  (0.003) |  | -0.17 (0.44) |
| Human density | 0.14 (0.51) | -0.16  (0.47) | -0.08 (0.72) | -0.17 (0.43) | -0.33 (0.11) | -0.18 (0.39) | 0.4 (0.06) | 0.37 ().07) | -0.17 (0.43) | 0.22 (0.28) | -0.16  (0.47) | -0.21  (0.33) | -0.17 (0.44) |  |

Table S15. Correlation results (*r* coefficients) for environmental and spatial predictors with antimicrobial resistance gene abundance in sediment samples (n=12) collected in 2019 (p-values in parentheses).

|  | *bla*_SHV_ | *ermB* | *intI1* | *intI3* | *mefE* | *mexB* | *strB* | *sul1* | *sul2* | *sul3* | *tet*(A) | *tet*(W) | Water pH | Water temp. | Conductivity | Animal Units | Human density |
| --- | --- | --- | --- | --- | --- | --- | --- | --- | --- | --- | --- | --- | --- | --- | --- | --- | --- |
| *bla*_SHV_ |  | 0.34 (0.17) | 0.66 (0.03) | -0.16 (0.45) | 0.06 (0.83) | 0.29 (0.26) | 0.34 (0.17) | 0.33 (0.18) | 0.24 (0.33) | 0.35 (0.15) | 0.62 (0.01) | 0.17 (0.50) | -0.15 (0.54) | -0.08 (0.76) | 0.16 (0.53) | 0.06 (0.81) | -0.26 (0.31) |
| *ermB* | 0.34 (0.17) |  | 0.36 (0.14) | 0.46 (0.06) | 0.31 (0.20) | 0.58 (0.01) | 0.60 (0.01) | 0.16 (0.52) | 0.46 (0.05) | 0.48 (0.04) | 0.48 (0.05) | 0.26 (0.29) | 0.12 (0.63) | -0.40 (0.1) | 0.38 (0.12) | 0.12  (0.65) | -0.26 (0.31) |
| *intI1* | 0.66 (0.03) | 0.36 (0.14) |  | 0.75 (0.0003) | 0.67 (0.002) | 0.45 (0.06) | 0.50 (0.04) | 0.05 (0.85) | 0.39 (0.11) | 0.53 (0.02) | 0.81 (4.62×10^-5^) | 0.65 (0.003) | 0.10 (0.68) | -0.47 (0.05) | 0.12 (0.65) | 0.11 (0.66) | -0.35 (0.16) |
| *intI3* | -0.16 (0.45) | 0.46 (0.06) | 0.75 (0.0003) |  | 0.72 (0.001) | 0.35 (0.16) | 0.60 (0.01) | 0.20 (0.43) | 0.63 (0.005) | 0.65 (0.004) | 0.59 (0.01) | 0.29 (0.25) | 0.08 (0.75) | -0.57 (0.01) | -0.07 (0.79) | -0.08 (0.75) | -0.03 (0.92) |
| *mefE* | 0.06 (0.83) | 0.31 (0.20) | 0.67 (0.002) | 0.72 (0.001) |  | 0.32 (0.19) | 0.37 (0.13) | -0.21 (0.41) | 0.34 (0.16) | 0.37 (0.13) | 0.45 (0.06) | 0.54 (0.02) | 0.16 (0.53) | -0.44 (0.07) | -0.13 (0.61) | -0.03 (0.90) | -0.18 (0.46) |
| *mexB* | 0.29 (0.26) | 0.58 (0.01) | 0.45 (0.06) | 0.35 (0.16) | 0.32 (0.19) |  | 0.21 (0.40) | -0.004 (0.99) | 0.12 (0.64) | 0.39 (0.10) | 0.56 (0.02) | 0.24 (0.34) | 0.11 (0.66) | -0.54 (0.02) | 0.25 (0.31) | -0.03 (0.89) | 0.06 (0.81) |
| *strB* | 0.34 (0.17) | 0.60 (0.01) | 0.50 (0.04) | 0.60 (0.01) | 0.37 (0.13) | 0.21 (0.40) |  | 0.22 (0.38) | 0.68 (0.002) | 0.37 (0.14) | 0.59 (0.009) | 0.47 (0.05) | 0.12 (0.64) | -0.57 (0.01) | 0.20 (0.43) | -0.06 (0.82) | -0.17 (0.51) |
| *sul1* | 0.33 (0.18)) | 0.16 (0.52) | 0.05 (0.85) | 0.20 (0.43) | -0.21 (0.41) | -0.004 (0.99) | 0.22 (0.38) |  | 0.34 (0.17) | 0.19 (0.46) | 0.36 (0.14) | -0.39 (0.11) | 0.24 (0.34) | -0.16 (0.53) | 0.20 (0.44) | 0.10 (0.70) | 0.34 (0.17) |
| *sul2* | 0.24 (0.33) | 0.46 (0.05) | 0.39 (0.11) | 0.63 (0.005) | 0.34 (0.16) | 0.12 (0.64) | 0.68 (0.002) | 0.34 (0.17) |  | 0.27 (0.28) | 0.34 (0.16) | 0.29 (0.24) | -0.13 (0.60) | -0.46 (0.06) | 0.20 (0.42) | 0.09 (0.74) | 0.09 (0.73) |
| *sul3* | 0.35 (0.15) | 0.48 (0.04) | 0.53 (0.02) | 0.65 (0.004) | 0.37 (0.13) | 0.39 (0.10) | 0.37 (0.14) | 0.19 (0.46) | 0.27 (0.28) |  | 0.44 (0.06) | 0.13 (0.62) | 0.42 (0.08) | -0.52 (0.03) | -0.14 (0.58) | -0.04 (0.86) | 0.19 (0.45) |
| *tet*(A) | 0.62 (0.01) | 0.48 (0.05) | 0.81 (4.62×10^-5^) | 0.59 (0.01) | 0.45 (0.06) | 0.56 (0.02) | 0.59 (0.009) | 0.36 (0.14) | 0.34 (0.16) | 0.44 (0.06) |  | 0.40 (0.1) | 0.15 (0.56) | -0.61 (0.01) | 0.37 (0.14) | 0.07 (0.78) | -0.11 (0.67) |
| *tet* (W) | 0.17 (0.50) | 0.26 (0.29) | 0.65 (0.003) | 0.29 (0.25) | 0.54 (0.02) | 0.24 (0.34) | 0.47 (0.05) | -0.39 (0.11) | 0.29 (0.24) | 0.13 (0.62) | 0.40 (0.1) |  | 0.11 (0.65) | -0.26 (0.29) | 0.1 (0.70) | 0.19 (0.44) | -0.47 (0.05) |
| Water pH | -0.15 (0.54) | 0.12 (0.63) | 0.10 (0.68) | 0.08 (0.75) | 0.16 (0.53) | 0.11 (0.66) | 0.12 (0.64) | 0.24 (0.34) | -0.13 (0.60) | 0.42 (0.08) | 0.15 (0.56) | 0.11 (0.65) |  | -0.20 (0.43) | -0.05 (0.83) | -0.05 (0.84) | 0.19 (0.44) |
| Water temperature | -0.08 (0.76) | -0.40 (0.1) | -0.47 (0.05) | -0.57 (0.01) | -0.44 (0.07) | -0.54 (0.02) | -0.57 (0.01) | -0.16 (0.53) | -0.46 (0.06) | -0.52 (0.03) | -0.61 (0.01) | -0.26 (0.29) | -0.20 (0.43) |  | 0.06 (0.83) | 0.39 (0.11) | -0.23 (0.36) |
| Conductivity | 0.16 (0.53) | 0.38 (0.12) | 0.12 (0.65) | -0.07 (0.79) | -0.13 (0.61) | 0.25 (0.31) | 0.20 (0.43) | 0.20 (0.44) | 0.20 (0.42) | -0.14 (0.58) | 0.37 (0.14) | 0.1 (0.70) | -0.05 (0.83) | 0.06 (0.83) |  | 0.58 (0.003) | -0.18 (0.48) |
| Animal Units | 0.06 (0.81) | 0.12  (0.65) | 0.11 (0.66) | -0.08 (0.75) | -0.03 (0.90) | -0.03 (0.89) | -0.06 (0.82) | 0.10 (0.70) | 0.09 (0.74) | -0.04 (0.86) | 0.07 (0.78) | 0.19 (0.44) | -0.05 (0.84) | 0.39 (0.11) | 0.58 (0.003) |  | -0.14 (0.59) |
| Human density | -0.26 (0.31) | -0.26 (0.31) | -0.35 (0.16) | -0.03 (0.92) | -0.18 (0.46) | 0.06 (0.81) | -0.17 (0.51) | 0.34 (0.17) | 0.09 (0.73) | 0.19 (0.45) | -0.11 (0.67) | -0.47 (0.05) | 0.19 (0.44) | -0.23 (0.36) | -0.18 (0.48) | -0.14 (0.59) |  |

Table S16. Correlation results (*r* coefficients) between antimicrobial resistance genes abundance and antimicrobial concentrations (ciprofloxacin, tetracycline, and sulfadimethoxine) for 2019 water samples (n=24). p-values in parentheses.

|  | Cipro. | Tetracy. | Sulfadimeth. | *bla*_SHV_ | *IMP-13* | *intI1* | *intI3* | *mexB* | *strB* | *sul1* | *sul2* | *tet*(A) |
| --- | --- | --- | --- | --- | --- | --- | --- | --- | --- | --- | --- | --- |
| Cipro. |  | -0.06  (0.79) | -0.08  (0.71) | 0.29  (0.17) | -0.14  (0.51) | -0.16  (0.45) | -0.17  (0.43) | 0.56  (0.004) | -0.12  (0.58) | -0.33  (0.12) | -0.30  (0.15) | -0.13  (0.54) |
| Tetracy. | -0.06  (0.79) |  | 0.18  (0.39) | -0.15  (0.48) | -0.11  (0.62) | -0.14  (0.52) | -0.11  (0.60) | -0.10  (0.89) | -0.09  (0.67) | -0.17  (0.42) | -0.12  (0.58) | -0.10  (0.64) |
| Sulfadimeth. | -0.08  (0.71) | 0.18  (0.39) |  | -0.17  (0.42) | -0.02  (0.93) | -0.05  (0.81) | 0.10  (0.65) | -0.02  (0.93) | -0.05  (0.80) | 0.04  (0.85) | 0.20  (0.36) | 0.34  (0.11) |
| *bla*_SHV_ | 0.29  (0.17) | -0.15  (0.48) | -0.17  (0.42) |  | 0.23  (0.29) | 0.35  0.10) | -0.16  (0.45) | 0.17  (0.43) | 0.24  (0.26) | 0.24  (0.25) | 0.40  (0.05) | 0.19  (0.37) |
| *imp13* | -0.14  (0.51) | -0.11  (0.62) | -0.02  (0.93) | 0.23  (0.29) |  | 0.44  (0.33) | 0.43  (0.04) | 0.56  (0.005) | -0.003  (0.99) | 0.05  (0.83) | -0.03  (0.88) | 0.52  (0.01) |
| *intI1* | -0.16  (0.45) | -0.14  (0.52) | -0.05  (0.81) | 0.35  (0.10) | 0.44  (0.03) |  | 0.30  (0.16) | 8.9x10^-5^  (1.0) | 0.26  (0.22) | 0.31  (0.14) | 0.20  (0.35) | 0.49  (0.01) |
| *intI3* | -0.16  (0.43) | -0.11  (0.60) | 0.10  (0.65) | -0.16  (0.45) | 0.43  (0.04) | 0.30  (0.16) |  | -0.03  (0.88) | 0.40  (0.06) | 0.24  (0.25) | 0.10  (0.66) | 0.21  (0.34) |
| *mexB* | 0.56  (0.004) | -0.03  (0.89) | -0.02  (0.93) | 0.17  (0.43) | 0.56  (0.005) | 8.9x10^-5^  (1.0) | -0.03  (0.88) |  | -0.33  (0.11) | -0.41  (0.05) | -0.54  (0.007) | 0.27  (0.20) |
| *strB* | -0.12  (0.58) | -0.09  (0.67) | -0.05  (0.80) | 0.24  (0.26) | -0.003  (0.99) | 0.26  (0.22) | 0.40  (0.06) | -0.33  (0.11) |  | 0.20  (0.36) | 0.52  (0.01) | 0.35  (0.01) |
| *sul1* | -0.32  (0.12) | -0.17  (0.42) | 0.04  (0.85) | 0.24  (0.25) | 0.05  (0.83) | 0.31  (0.14) | 0.24  (0.25) | -0.41  (0.05) | 0.20  (0.36) |  | 0.65  (0.00) | 0.25  (0.23) |
| *sul2* | -0.30  (0.15) | -0.12  (0.58) | 0.20  (0.36) | 0.40  (0.05) | -0.03  (0.88) | 0.20  (0.35) | 0.10  (0.66) | -0.54  (0.01) | 0.52  (0.01) | 0.65  (0.00) |  | 0.40  (0.05) |
| *tet*(A) | -0.131  (0.54) | -0.10  (0.64) | 0.34  (0.11) | 0.19  (0.37) | 0.52  (0.01) | 0.50  (0.01) | 0.20  (0.34) | 0.27  (0.20) | 0.35  (0.10) | 0.25  (0.23) | 0.40  (0.05) |  |

Table S17. Correlations results (*r* coefficients) between antimicrobial resistance genes abundance and antimicrobial concentrations (tetracycline, and sulfadimethoxine) for 2019 sediment samples (n=23).

|  | Tetrac. | Sulfadim. | *bla*_SHV_ | *ermB* | *intI1* | *intI3* | *mefE* | *mexB* | *strB* | *sul1* | *sul2* | *sul3* | *tet*(A) | *tet*(W) |
| --- | --- | --- | --- | --- | --- | --- | --- | --- | --- | --- | --- | --- | --- | --- |
| Tetrac. |  | 0.13  (0.56) | 0.32  (0.13) | 0.39  (0.07) | 0.27  (0.21) | 0.37  (0.08) | -0.11  (0.63) | 0.39  (0.07) | 0.16  (0.47) | 0.21  (0.33) | 0.29  (0.19) | 0.12  (0.19) | 0.43  (0.57) | 0.26  (0.23) |
| Sulfadim. | 0.13  (0.56) |  | -0.04  (0.85) | 0.42  (0.05) | 0.22  (0.32) | 0.03  (0.89) | -0.03  (0.86) | 0.14  (0.51) | 0.04  (0.86) | -0.25  (0.26) | 0.14  (0.53) | 0.31  (0.15) | 0.34  (0.12) | 0.48  (0.02) |
| *bla*_SHV_ | 0.32  (0.13) | -0.04  (0.85) |  | 0.30  (0.16) | 0.72  (0.00) | 0.55  (0.00) | 0.03  (0.89) | 0.58  (0.00) | -0.23  (0.29) | 0.50  (0.02) | 0.40  (0.06) | 0.29  (0.19) | 0.62  (0.00) | -0.15  (0.50) |
| *ermB* | 0.39  (0.07) | 0.42  (0.05) | 0.30  (0.16) |  | 0.44  (0.04) | 0.37  (0.08) | 0.26  (0.24) | 0.46  (0.03) | 0.24  (0.27) | 0.36  (0.09) | 0.37  (0.08) | 0.83  (1x10^-06^) | 0.50  (0.02) | 0.36  (0.09) |
| *intI1* | 0.27  (0.21) | 0.22  (0.32) | 0.72  (0.00) | 0.44  (0.04) |  | 0.63  (0.00) | 0.25  (0.25) | 0.72  (9.11x10^-05^) | 0.04  (0.85) | 0.46  (0.03) | 0.53  (0.01) | 0.36  (0.09) | 0.74  (6.32x10^-05^) | 0.28  (0.19) |
| *intI3* | 0.37  (0.08) | 0.03  (0.89) | 0.55  (0.00) | 0.37  (0.08) | 0.63  (0.00) |  | 0.19  (0.37) | 0.57  (0.00) | -0.11  (0.61) | 0.38  (0.07) | 0.30  (0.17) | 0.33  (0.13) | 0.57  (0.00) | 0.28  (0.19) |
| *mefE* | -0.11  (0.63) | -0.04  (0.86) | 0.03  (0.89) | 0.26  (0.24) | 0.25  (0.25) | 0.19  (0.37) |  | -0.09  (0.68) | 0.16  (0.47) | -0.09  (0.67) | 0.19  (0.39) | 0.33  (0.13) | -0.00  (0.99) | 0.05  (0.83) |
| *mexB* | 0.39  (0.07) | 0.14  (0.51) | 0.58  (0.00) | 0.46  (0.03) | 0.72  (9.11x10^-05^) | 0.57  (0.00) | -0.09  (0.68) |  | -0.03  (0.90) | 0.50  (0.01) | 0.43  (0.04) | 0.36  (0.10) | 0.70  (0.00) | 0.33  (0.13) |
| *strB* | 0.16  (0.47) | 0.04  (0.86) | -0.23  (0.29) | 0.24  (0.27) | 0.04  (0.85) | -0.11  (0.61) | 0.16  (0.47) | -0.03  (0.90) |  | 0.04  (0.84) | 0.27  (0.21) | 0.19  (0.39) | 0.03  (0.90) | 0.158116 |
| *sul1* | 0.21  (0.33) | -0.25  (0.26) | 0.50  (0.02) | 0.36  (0.09) | 0.46  (0.03) | 0.38  (0.07) | -0.09  (0.67) | 0.50  (0.01) | 0.04  (0.84) |  | 0.29  (0.17) | 0.21  (0.34) | 0.27  (0.21) | -0.27  (0.21) |
| *sul2* | 0.29  (0.19) | 0.14  (0.53) | 0.40  (0.06) | 0.37  (0.08) | 0.53  (0.01) | 0.30  (0.17) | 0.19  (0.39) | 0.43  (0.04) | 0.27  (0.21) | 0.29  (0.17) |  | 0.34  (0.12) | 0.35  (0.10) | -0.00  (1.0) |
| *sul3* | 0.12  (0.57) | 0.31  (0.15) | 0.29  (0.19) | 0.83  (1.04x10^-06^) | 0.36  (0.09) | 0.33  (0.13) | 0.33  (0.13) | 0.36  (0.10) | 0.19  (0.39) | 0.21  (0.34) | 0.34  (0.12) |  | 0.30  (0.17) | 0.22  (0.31) |
| *tet*(A) | 0.43  (0.04) | 0.34  (0.12) | 0.62  (0.00) | 0.50  (0.02) | 0.74  (6.32x10^-05^) | 0.57  (0.00) | -0.00  (1.0) | 0.70  (0.00) | 0.03  (0.90) | 0.27  (0.21) | 0.35  (0.10) | 0.30  (0.17) |  | 0.53  (0.10) |
| tet(W) | 0.26  (0.23) | 0.48  (0.02) | -0.15  (0.50) | 0.36  (0.09) | 0.28  (0.19) | 0.28  (0.19) | 0.05  (0.83) | 0.33  (0.13) | 0.16  (0.47) | -0.27  (0.21) | -0.00  (1.0) | 0.22  (0.31) | 0.53  (0.10) |  |

Table S18. Arithmetic mean and standard deviation (in parentheses) for the concentration of antimicrobials in water samples in 2019 in waterbodies with direct wastewater discharge (n=4).

| Site | Ciprofloxacin | Tetracycline | Sulfadimethoxine |
| --- | --- | --- | --- |
| Upstream | 1.61 (0.68) | 2.67 (2.76) | 6.47 (7.56) |
| Wastewater site | 58.3 (113.0) | 5.12 (4.76) | 7.83 (15.4) |
| Downstream | 1.61 (0.68) | 2.58 (2.58) | 4.95 (9.68) |

Table S19. Arithmetic mean and standard deviation (in parentheses) for the concentration of antimicrobials in sediment samples in 2019 in waterbodies with wastewater discharge (n=3). Na: not applicable.

| Site | Tetracycline | Sulfadimethoxine |
| --- | --- | --- |
| Upstream site 1 | 9.81 (8.75) | 181 (241) |
| Wastewater site | 26.7 (31.5) | 248 (344) |
| Downstream site 1 | 7.96 (6.15) | 114 (167) |
| Downstream site 2 | 5.67 (2.05) | 226 (309) |
| Downstream site 3 | 4.22 (na) | 7.36 (na) |

**Supplementary Methods**

**Sample collection**

Samples were collected by following different procedures depending on the analytical purpose. For antimicrobial analyses, water samples (17 samples in 2018 and 35 samples from 19 different waterbodies in 2019) were collected at elbow depth in a pre-combusted (400 ⁰C, 4 hr) 1-gallon jug which was covered with a towel to avoid sunlight until placed in a cooler with ice. After returning to the laboratory, water samples were stored at 1 ⁰C and processed within seven days. For ARG determination in water samples (38 samples from 22 different waterbodies of which 14 sites were sampled twice in 2018, and 24 samples from 10 different waterbodies in 2019), samples were collected according to a previously published method^1^. Briefly, water samples were filtered through a REXEED 25S ultrafiltration membrane filter (Asahi Kasei, Tokyo, Japan) in the field (average sample volume: 37.6±2.1 L; arithmetic mean and one standard deviation). Filters were transported back to the laboratory on ice and stored at 4 °C for no more than 48 hr.

Sediment samples were collected from the bottom of each waterbody using an Ekman dredge. For antimicrobial analyses, sediment samples (none collected in 2018 and 31 from 17 different waterbodies in 2019) were transferred to a glass container, and for ARG (33 samples from 20 different waterbodies in 2018 and 30 samples from 12 different waterbodies in 2019), a sub-sample was taken from the dredge using a sterile scoopula and placed into 2 mL centrifuge tubes. Samples were then placed in a cooler with ice until being transported back to the laboratory.

A water quality sonde (Hatch Hydrolab Quanta, Hydrolab Corporation, Texas, USA) was used to measure water temperature, pH, and specific conductance during the 2019 field season. See Supplementary Table S22 for the Global Positioning System (GPS) coordinates.

**Laboratory Methods**

***Antimicrobials***

*Chemicals:* Ofloxacin (98%), amoxicillin (96%), penicillin G sodium salt (98%), and penicillin V potassium salt (>95%) were obtained from Acros Organics. Sulfamethoxazole (99%), chlortetracycline hydrochloride (92.9%), and nalidixic acid (>99%) were bought from Chem Impex International Inc. while tetracycline hydrochloride (98%) was obtained from Research Products International. Tylosin tartrate (≥95%), sulfadimethoxine (≥98.5%), trimethoprim (≥95%), doxycycline hyclate (≥98.0%), norfloxacin (≥98.0%), lincomycin hydrochloride (≥95%), roxithromycin (≥90%), sulfapyridine (≥99%), carbadox, enrofloxacin (≥98%), oxytetracycline hydrochloride (≥95%), sulfachloropyridazine (≥98.0%), erythromycin A dihydrate (VETRANAL), simeton (PESTANAL), ^13^C_6_-sulfamethoxazole (>99.5%), and sulfuric acid (>95%, ACS grade) were purchased from Sigma-Aldrich. Sulfadiazine (98.0%), sulfamethazine (>98.0%), azithromycin dihydrate (>98.0%), clarithromycin (>98%), and ciprofloxacin (>98%) were obtained from Tokyo Chemical Industry. Cambridge Isotopes supplied ^13^C_2_-erythromycin (~90%) and ^13^C_6_-sulfamethazine (99%). Clinafloxacin (≥95%) and demeclocycline hydrochloride (≥93%) were purchased from Santa Cruz Biotechnology, Inc.

Citric acid monohydrate (ACS grade) was bought from Mallinckrodt Chemicals and sodium phosphate dibasic anhydrous (ACS grade) was purchased from J.T. Baker. Industrial grade nitrogen was supplied by Matheson Gas. Fisher Scientific produced Ottawa sand, ethyl acetate (HPLC grade), acetone (HPLC grade), acetonitrile (HPLC grade), methanol (HPLC grade and Optima® LC-MS), and formic acid (ACS grade and Optima® LC-MS). The 1000 μΩ/cm conductivity standard and nitric acid (68-70%, ACS grade) was bought from VWR and ultrapure water (18.2 MΩ-cm) came from the Millipore Simplicity UV purification system. Ethylenediaminetetraacetic acid (EDTA) disodium salt dihydrate (>99%) was obtained from Avocado Research Chemicals and phosphoric acid (85%, ACS grade) was bought from Chempure.

Water samples were filtered with a pre-combusted glass fiber filter and then adjusted to pH 3 with sulfuric acid. The samples were split into triplicates and all spiked with 200 ng of surrogates (^13^C_6_-sulfamethazine, nalidixic acid, and demeclocycline) and 10 mL of 0.1 mg/mL EDTA. One sample was spiked with 100 ng of all of the antimicrobials tested. Samples were then stored at 1 ⁰C overnight to equilibrate. Solid phase extraction (SPE) was then performed on the samples using HLB cartridges (Waters 6 cc, 200 mg, 30 μm). Cartridges were cleaned with 3 mL of MilliQ water, methanol, MilliQ water, and then pH 3 MilliQ water. Samples were passed through using fluoropolymer tubing (Saint-Gobain Chemofluor), previously cleaned with water and methanol, at a maximum rate of 15 mL/minute and a maximum pressure of 15 mmHg. Tubing was never reused. The sample was loaded, passed through, and then the cartridges were cleaned with 3 mL of MilliQ water. Cartridges were dried under vacuum for 1 minute and then eluted under vacuum pulses into glass test tubes with 13 mL of methanol. Eluents were spiked with 100 ng of internal standards (clinafloxacin, ^13^C_2_-erythromycin, simeton, and ^13^C_6_-sulfamethoxazole) for samples collected between June 25, 2019 and July 22, 2019 and 20 ng of internal standards for samples collected between July 31, 2019 and August 27, 2019. The eluent was evaporated to dryness under a gentle stream of nitrogen in a 40⁰C water bath. Once dry, samples were resuspended in 200 μL of 20 mM ammonium acetate and then filtered with a 0.4 μm GHP syringe filter (Whatman, 13 mm) into an HPLC vial.

Sediment samples were analyzed by weighing out 0.5 g of freeze-dried sediment into a 15 mL plastic centrifuge tube (soaked overnight in a 2% nitric acid bath prior)^2^. Sediment samples were analyzed in two parts: one sample and one test for spike and recovery. Both received 200 ng of surrogates (^13^C_6_-sulfamethazine, nalidixic acid, and demeclocycline) and 2.5 g of Ottawa sand while only the one test for spike and recovery also received 100 ng of antimicrobials. The same antimicrobials were analyzed in both water and sediment samples except for lincomycin, amoxicillin, penicillin V, and penicillin G which were not analyzed in sediment due to low to nonexistent recoveries in sediment processing. The mixture was vortexed and allowed to stand for 30 minutes to equilibrate. Then, 10 mL of 30:20:50 ACN:MeOH: 0.1 M EDTA/0.08 M disodium phosphate/0.06 M citrate buffer (pH 4, v/v/v) solution was added to each sample. The samples were vortexed for 30 seconds, placed in an ultrasonic bath for 10 minutes (Bransonic, 2210R-DTH, 50/60 kHz), and centrifuged for 10 minutes (3300 rpm). This step was repeated twice more, and the extracts were combined. The methanol and acetonitrile in the extracts were removed using a rotary evaporator (35 °C). Extracts were then diluted to 400 mL with MilliQ and adjusted to pH 4 with phosphoric acid.

Solid phase extraction was then performed on the aqueous solutions with HLB cartridges that were cleaned with 10 mL of methanol and 10 mL of water. Samples were transferred using fluoropolymer tubing (Saint-Gobain Chemofluor) that was cleaned with methanol and water prior to use. Once completed, the cartridges were washed with 6 mL of 40:60 methanol:water and then eluted with 13 mL of methanol. Samples were spiked with 20 ng of internal standard (clinafloxacin, ^13^C_2_-erythromycin, simeton, and ^13^C_6_-sulfamethoxazole) and evaporated to dryness with a vacufuge (Eppendorf vacufuge plus, 1400 rpm). The samples were reconstituted with 200 μL of 20 mM ammonium acetate and filtered with a 0.45 μm GHP syringe filter (Whatman, 13 mm) into an HPLC vial.

*HPLC-MS/MS Analysis*: Both water and sediment samples were analyzed on a Dionex Ultimate 3000 with a Thermo Scientific TSQ Vantage triple quadrupole tandem mass spectrometer in positive ESI mode. Samples were analyzed by three separate methods: one for fluoroquinolones and tetracyclines, one for macrolides and β-lactams, and one for sulfonamides and other compounds^2^. The specific conditions are listed in Supplementary Table S23, the quantification and confirmation values are listed in Supplementary Table S24, and the gradients for the three methods are listed in Supplementary Table S25. Normal values for the mass spectrometer were: 0.010 s scan time, 0.010 scan width, 0.7 Q1/Q2, 3300 V spray voltage, 50 psi sheath gas pressure, 300 ⁰C capillary temperature, 1.5 mTorr collision pressure, 0 V declustering voltage, and 95 S lens.

For the sediment samples, additional cleaning was required prior to sample analysis on the HPLC-MS/MS. When analyzing the sediment samples, the fluoroquinolone peaks disappeared from both the standards and samples. After investigating various fixes, it was determined that a substance in the sediment samples was sorbing to the column which was then sorbing the fluoroquinolones. So prior to the analysis, the column was cleaned with 50:50 20 mM EDTA:MeOH for 1 hour at 100 μL/minute followed by 50:50 MeOH:MilliQ water for 30 minutes at 100 μL/minute. If the fluroquinolone peak areas were still decreasing, an additional cleaning of the following solvents at 500 μL/min for 7 minutes each was performed: 20:80 isopropanol:water, tetrahydrofuran, dichloromethane, hexane, 20:80 isopropanol:water. The isopropanol:water mixtures started at 20:80 and slowly increased to 50:50 while watching the pressure. Additionally, only 20 injections were done before repeating the cleaning procedure to avoid buildup on the column.

Limits of detection (LOD) were determined as three times the method blank concentration and limits of quantification (LOQ) were set as ten times the method blank concentration minus one method blank concentration to account for noise. Samples were recovery corrected, but LODs and LOQs were not.

Table S20. Global positioning system (GPS) coordinates (latitude, longitude) for the sampling sites in 2018 and 2019.

| Site Name | Latitude | Longitude |
| --- | --- | --- |
| Baptism River | 47.373004 | -91.228817 |
| Blue Earth River | 44.131689 | -94.045946 |
| Cedar Lake | 47.96364 | -91.77774 |
| Crosby Wastewater treatment plant | 46.48416 | -93.96215 |
| Crosslake | 46.670516 | -94.112961 |
| Fenske Lake | 47.9967 | -91.91271 |
| Florida Slough Lake | 45.2471932 | -95.0742023 |
| Lake Andrew | 45.3136511 | -95.0468711 |
| Lake Bemidji | 47.46351981 | -94.8841084 |
| Lake Benton | 44.289091 | -96.253639 |
| Lake Edward | 46.50913 | -94.16067 |
| Lake Emily | 44.308533 | -93.917826 |
| Lake Harriet | 44.922059 | -93.30584 |
| Lake Ida | 45.98141008 | -95.39980317 |
| Lake Owasso | 45.037427 | -93.121035 |
| Lake Shetek | 44.168632 | -95.727386 |
| Lake St. Anna | 45.745299 | -94.616541 |
| Lake Wakanda | 45.05912 | -94.969355 |
| Lake Winona | 45.87517912 | -95.40514187 |
| Little Rabbit Lake | 46.46411 | -94.05332 |
| Little Rock Lake | 45.7450285 | -94.1620979 |
| Long Lake | 45.704821 | -94.713868 |
| Long Prairie River | 45.98007497 | -95.3120848 |
| Medicine Lake | 45.010519 | -93.422811 |
| Minnesota River | 44.82723 | -93.23018 |
| Mississippi River | 46.50189 | -94.08534 |
| Ocheda Lake | 43.55762 | -95.634086 |
| Otsego Creek | 45.262316 | -93.654976 |
| Pepin Lake | 45.910335 | -94.648683 |
| Pine River | 46.57242 | -94.02843 |
| Sakatah Lake | 44.224599 | -93.538933 |
| Serpent Lake | 46.4779 | -93.94057 |
| St. Croix River | 45.01929 | -92.76342 |
| St. Louis River | 47.37198 | -92.57586 |
| White Iron Lake | 47.89115 | -91.77623 |

Table S21. LC-MS/MS parameters.

| Parameter | Method |
| --- | --- |
| Stationary Phase | Waters Xselect CSH C18 (3.5 μm, 130 Å, 50x2.1 mm) |
| Flow rate | 500 μL/minute |
| Temperature | 35 ⁰C |
| Injection Volume | 8 μL |
| Flow Diverted to Waste | 0-1.5 min, 5.5-20 min |
| Mobile Phase A | 0.1% formic acid in water |
| Mobile Phase B | 0.1% formic acid in methanol |

Table S22. Single reaction monitoring quantification and confirmation transitions and collision energy (CE) for all analytes.

| Analyte | Parent Ion (m/z) | Product Ion (m/z) | CE (V) | Quantification or Confirmation |
| --- | --- | --- | --- | --- |
| Sulfonamides | | | | |
| Sulfapyridine | 250.10 | 156.00 | 17 | quantification |
|  | 250.10 | 108.05 | 25 | confirmation |
| Sulfadiazine | 251.05 | 156.00 | 15 | quantification |
|  | 251.05 | 108.05 | 24 | confirmation |
| Sulfamethoxazole | 254.05 | 92.10 | 29 | quantification |
|  | 254.05 | 108.00 | 24 | confirmation |
| Sulfamethazine | 279.05 | 186.00 | 17 | quantification |
|  | 279.05 | 156.00 | 20 | confirmation |
| Sulfachloropyridazine | 285.00 | 156.06 | 21 | quantification |
|  | 285.00 | 92.05 | 35 | confirmation |
| Sulfadimethoxine | 311.10 | 156.06 | 21 | quantification |
|  | 311.10 | 92.05 | 35 | confirmation |
| ^13^C_6_-sulfamethoxazole (internal standard) | 260.05 | 98.10 | 32 | quantification |
|  | 260.05 | 114.10 | 27 | confirmation |
| ^13^C_6_-sulfamethazine (surrogate) | 285.05 | 186.00 | 22 | quantification |
|  | 285.05 | 123.00 | 20 | confirmation |
| Fluoroquinolones | | | | |
| Norfloxacin | 320.10 | 276.10 | 17 | quantification |
|  | 320.10 | 302.10 | 21 | confirmation |
| Ciprofloxacin | 332.10 | 231.05 | 35 | quantification |
|  | 332.10 | 314.10 | 21 | confirmation |
| Enrofloxacin | 360.10 | 245.10 | 25 | quantification |
|  | 360.10 | 316.15 | 19 | confirmation |
| Ofloxacin | 362.10 | 261.10 | 28 | quantification |
|  | 362.10 | 318.10 | 19 | confirmation |
| Clinafloxacin  (Internal Standard) | 366.10 | 348.00 | 20 | confirmation |
|  | 366.10 | 305.00 | 22 | quantification |
| Nalidixic Acid  (Surrogate) | 233.15 | 187.00 | 27 | confirmation |
|  | 233.15 | 104.05 | 40 | quantification |
| Tetracyclines | | | | |
| Tetracycline | 445.10 | 410.10 | 19 | quantification |
|  | 445.10 | 427.05 | 11 | confirmation |
| Doxycycline | 445.10 | 321.05 | 31 | quantification |
|  | 445.10 | 428.15 | 18 | confirmation |
| Oxytetracycline | 461.10 | 426.10 | 17 | quantification |
|  | 461.10 | 443.10 | 12 | confirmation |
| Chlortetracycline and degradation products | 479.05 | 462.10 | 20 | quantification |
|  | 479.05 | 444.10 | 17 | confirmation |
|  | 481.05 | 464.10 | 20 | quantification |
|  | 481.05 | 446.10 | 30 | confirmation |
| Demeclocycline  (Surrogate) | 465.10 | 448.05 | 20 | quantification |
|  | 465.10 | 430.05 | 17 | confirmation |
| Macrolides | | | | |
| Azithromycin | 375.26 | 83.08 | 24 | quantification |
|  | 375.26 | 158.15 | 24 | confirmation |
| Clarithromycin | 748.51 | 158.04 | 30 | quantification |
|  | 748.51 | 590.49 | 17 | confirmation |
| Erythromycin | 734.4 | 158.15 | 35 | quantification |
|  | 734.4 | 576.35 | 15 | confirmation |
| Erythromycin-H_2_O | 716.45 | 158.15 | 35 | quantification |
|  | 716.45 | 558.35 | 15 | confirmation |
| Roxithromycin | 837.45 | 158.10 | 35 | quantification |
|  | 837.45 | 679.45 | 20 | confirmation |
| Tylosin | 916.45 | 174.10 | 40 | quantification |
|  | 916.45 | 772.45 | 30 | confirmation |
| ^13^C_2_-erythomycin  (Internal Standard) | 736.40 | 160.15 | 35 | quantification |
|  | 736.40 | 578.35 | 20 | confirmation |
| ^13^C_2_-erythromycin-H_2_O (Internal Standard) | 718.40 | 160.15 | 35 | quantification |
|  | 718.40 | 560.35 | 20 | confirmation |
| Non-Categorized | | | | |
| Carbadox | 263.10 | 130.05 | 22 | quantification |
|  | 263.10 | 231.05 | 13 | confirmation |
| Trimethoprim | 291.10 | 230.10 | 23 | quantification |
|  | 291.10 | 123.05 | 24 | confirmation |
| Lincomycin | 407.30 | 126.10 | 35 | quantification |
|  | 407.30 | 359.20 | 18 | confirmation |
| Simeton  (Internal Standard) | 198.20 | 68.10 | 33 | quantification |
|  | 198.20 | 100.10 | 27 | confirmation |
| β-Lactams | | | | |
| Amoxicillin | 366.15 | 114.01 | 22 | quantification |
|  | 366.15 | 349.20 | 8 | confirmation |
| Penicillin G | 335.11 | 160.06 | 12 | quantification |
|  | 335.11 | 176.11 | 15 | confirmation |
| Penicillin V | 351.12 | 114.05 | 33 | quantification |
|  | 351.12 | 160.07 | 15 | confirmation |

Table S23. LC-MS/MS gradients for water and sediment samples.

| Sulfonamides, non-categorized, macrolides, and β-lactams | | Tetracyclines and Fluoroquinolones | |
| --- | --- | --- | --- |
| Time (min) | % B | Time (min) | % B |
| 0.0 | 0 | 0.0 | 0 |
| 5.5 | 100 | 0.5 | 0 |
| 7.5 | 100 | 4.0 | 40 |
| 8.0 | 0 | 7.0 | 100 |
| 20.0 | 0 | 9.0 | 100 |
| -- | -- | 10.0 | 0 |
| -- | -- | 20.0 | 0 |

***Antimicrobial resistance genes***

Filters obtained with the REXEED 25S (Asahi Kasei, Tokyo, Japan) ultrafiltration system in the field were back flushed using 500 mL of sterile back flushing solution (0.01 M phosphate-buffered saline [pH 7.4] with 0.01% [v/v] Tween 80, and 0.001% [v/v] Y-30 antifoam emulsion). Polyethylene glycol (final concentration = 8% [w/v]), beef extract (final concentration = 1% [w/v]), and sodium chloride (final concentration = 0.2 M) were added to the back flushed solution, which was mixed for one hour at 4 °C and then quiescently stored at 4 °C for 24 hr. The back flushed solution was then centrifuged and decanted to concentrate the biomass. TE buffer (10×, pH 8.0) was used to resuspend the concentrated biomass. The final concentrated solution (average volume = 9.6 ± 5.3 mL) was stored at -20°C until DNA extraction.

Specific Target Amplification (STA): Briefly, STA of the standards and the samples was performed using SsoFast™ EvaGreen® Supermix with Low ROX (Bio-Rad, Hercules, CA, USA), targeting all the genes except for 16S rRNA gene. For STA, a thermal cycling protocol of 600 sec at 95 °C (initial denaturation) followed by 17 cycles of 15 sec at 95°C and 240 sec at 60 °C was used. Pre-amplified standards and samples were stored at 4°C for no more than 24 hours before MF-qPCR was performed. 192.24 Dynamic Array™ IFC for Gene Expression was used as manufacturer’s instruction (Fluidigm, South San Francisco, CA, USA) with the thermal cycling protocol of 60 sec at 95 °C (initial denaturation) followed by 40 cycles of 5 sec at 96 °C and 20 sec at 60 °C.

Table S24. Assay sequences and reasons for gene selection for Microfluidic-qPCR (MF- qPCR).

| Target Gene | Reason for  Gene Selection | Assay Sequence (5’🡪3’) | Reference |
| --- | --- | --- | --- |
| 16S rRNA | Total Bacterial Quantification | F: CCT ACG GGA GGC AGC AG  R: ATT ACC GCG GCT GCT GG | 3 |
| *ampC* | Ampicillin Resistance | F: CCT CTT GCT CCA CAT TTG CT  R: ACA ACG TTT GCT GTG TGA CG | 4 |
| *bla*_OXA_ | ß-lactams Resistance | F: TGA TGA TTG TCG AAG CCA AA  R: GCC TGT AGG CCA CTC TAC CC | 5 |
| *bla*_SHV_ | ß-lactams Resistance | F: AAC GGA ACT GAA TGA GGC GCT  R: TCC ACC ATC CAC TGC AGC AGC T | 6 |
| *ereB* | Erythromycin Resistance | F: TCT GCA TTA TGC CAA CGG TA  R: TCT GCT CAC TTT GTG GGT TTT | 4 |
| *ermB* | Erythromycin Resistance | F: GAT ACC GTT TAC GAA ATT GG  R: GAA TCG AGA CTT GAG TGT GC | 7 |
| *ermF* | Erythromycin Resistance | F: CGA CAC AGC TTT GGT TGA AC  R: GGA CCT ACC TCA TAG ACA AG | 8 |
| *IMP-13* | ß-lactams Resistance | F: AGG AGC GGC TTT ACC TGA TT  R: CGC TCC ACA AAC CAA TTG AC | 4 |
| *intI1* | Class 1 Integrase | F: CCT CCC GCA CGA TGA TC  R: TCC ACG CAT CGT CAG GC | 9 |
| *intI2* | Class 2 Integrase | F: GAC GGC TAC CCT CTG TTA TCT C  R: TGC TTT TCC CAC CCT TAC C | 10 |
| *intI3* | Class 3 Integrase | F: GGA TGT CTG TGC CTG CTT G  R: GCC ACC ACT TGT TTG AGG A | 10 |
| *mefE* | Macrolide Resistance | F: CCT GCA AAT GGC GAT TAT TT  R: AAT AGC AAG CAC TGC ACC AG | 4 |
| *mexB* | Multi-drug Efflux Pump | F: GTG TTC GGC TCG CAG TAC TC  R: AAC CGT CGG GAT TGA CCT TG | 11 |
| *qacF* | Quaternary Ammonia Disinfectant Resistance | F: TGG CTG TTT CAA TCT TTG GC  R: GCC CAT ACA GCG TAA GCA AT | 4 |
| *qnrA* | Quinolone Resistance | F: AGG ATT TCT CAC GCC AGG ATT  R: CCG CTT TCA ATG AAA CTG CA | 12 |
| *strB* | Streptomycin Resistance | F: CGC AGT TCA TCA GCA ATG TC  R: GCC TGT TTT TCC TGC TCA TT | 4 |
| *sul1* | Sulfonamide Resistance | F: CCG TTG GCC TTC CTG TAA AG  R: TTG CCG ATC GCG TGA AGT | 13 |
| *sul2* | Sulfonamide Resistance | F: GAC AGT TAT CAA CCC GCG AC  R: GTC TTG CAC CGA ATG CAT AA | 4 |
| *sul3* | Sulfonamide Resistance | F: TCC GTT CAG CGA ATT GGT GCA G  R: TTC GTT CAC GCC TTA CAC CAG C | 14 |
| *tet*(A) | Tetracycline Resistance | F: GCT ACA TCC TGC TTG CCT TC  R: CAT AGA TCG CCG TGA AGA GG | 15 |
| *tet*(M) | Tetracycline Resistance | F: GTG GAC AAA GGT ACA ACG AG  R: CGG TAA AGT TCG TCA CAC AC | 15 |
| *tet*(W) | Tetracycline Resistance | F: GAG AGC CTG CTA TAT GCC AGC  R: GGG CGT ATC CAC AAT GTT AAC | 16 |
| *tet*(X) | Tetracycline Resistance | F: AGC CTT ACC AAT GGG TGT AAA  R: TTC TTA CCT TGG ACA TCC CG | 17 |
| *vanA* | Vancomycin Resistance | F: GTA GGC TGC GAT ATT CAA AGC  R: CGA TTC AAT TGC GTA GTC CAA | 18 |

**Data analyses**

One animal unit (AU) is defined as 1,000 pounds based on the average animal weights^19^. One AU is equivalent to one cow, 2.5 pigs, 100 chickens (broilers or layers), 10 sheep, 10 goats, and 55 turkeys^19^.

Table S25. Kernel interpolation parameters and results for ciprofloxacin, tetracycline, and sulfadimethoxine for the 2019 averaged water samples across the same waterbody (n=19).

|  | Ciprofloxacin | Tetracycline | Sulfadimethoxine |
| --- | --- | --- | --- |
| Kernel function | Gaussian | Gaussian | Gaussian |
| Output surface type | Prediction | Prediction | Prediction |
| Order of polynomial | 1 | 1 | 1 |
| Ridge | 50 | 50 | 50 |
| Bandwidth (m) | 117710.90 | 139093.48 | 231098.65 |
| Neighborhood smoothing factor | 0.2 | 0.2 | 0.2 |
| Neighborhood radius (m) | 117710.90 | 139093.48 | 231098.65 |
| Root-Mean-Square | 26.68 | 93.53 | 13.12 |
| Mean Standardized | -0.08 | 0.11 | 0.13 |
| Root-Mean-Square Standardized | 1.05 | 1.34 | 1.23 |
| Average Std Error | 26.06 | 65.17 | 10.33 |

Table S26. Kernel interpolation parameters and results for tetracycline, and sulfadimethoxine for the 2019 averaged sediment samples across the same waterbody (n=17).

|  | Tetracycline | Sulfadimethoxine |
| --- | --- | --- |
| Kernel function | Gaussian | Gaussian |
| Output surface type | Prediction | Prediction |
| Order of polynomial | 1 | 1 |
| Ridge | 50 | 50 |
| Bandwidth (m) | 2.30 | 1.64 |
| Neighborhood smoothing factor | 0.2 | 0.2 |
| Neighborhood radius (m) | 2.30 | 1.64 |
| Root-Mean-Square | 96.09 | 189.87 |
| Mean Standardized | -0.04 | 0.07 |
| Root-Mean-Square Standardized | 0.93 | 0.89 |
| Average Std Error | 116.75 | 231.30 |

Table S27. Kernel interpolation parameters and results for three antimicrobial resistance genes (ARG) for the 2019 averaged water samples across waterbodies (n=10).

|  | *bla*_SHV_ | *sul1* | *tet*(A) |
| --- | --- | --- | --- |
| Kernel function | Gaussian | Gaussian | Gaussian |
| Output surface type | Prediction | Prediction | Prediction |
| Order of polynomial | 1 | 1 | 1 |
| Ridge | 50 | 50 | 50 |
| Bandwidth (m) | 1.48 | 1.03 | 2.16 |
| Neighborhood smoothing factor | 0.2 | 0.2 | 0.2 |
| Neighborhood radius (m) | 1.48 | 1.03 | 2.16 |
| Root-Mean-Square | 0.65 | 0.74 | 0.18 |
| Mean Standardized | -0.08 | NaN | -0.02 |
| Root-Mean-Square Standardized | 0.94 | NaN | 1.00 |
| Average Std Error | 0.69 | - | 0.18 |

NaN: Not a Number

-: there is no value for it

Table S28. Kernel interpolation parameters and results for antimicrobial resistance genes (ARG) for the 2019 averaged sediment samples across waterbodies (n=20).

|  | *bla*_SHV_ | *sul1* | tet*(A)* |
| --- | --- | --- | --- |
| Kernel function | Gaussian | Gaussian | Gaussian |
| Output surface type | Prediction | Prediction | Prediction |
| Order of polynomial | 1 | 1 | 1 |
| Ridge | 50 | 50 | 50 |
| Bandwidth (m) | 1.28 | 1.92 | 1.21 |
| Neighborhood smoothing factor | 0.2 | 0.2 | 0.2 |
| Neighborhood radius (m) | 1.28 | 1.92 | 1.21 |
| Root-Mean-Square | 1.11 | 0.93 | 1.20 |
| Mean Standardized | -0.49 | -0.12 | -0.59 |
| Root-Mean-Square Standardized | 1.86 | 1.33 | 2.29 |
| Average Std Error | 0.54 | 0.71 | 0.48 |

**Supplementary Figures**

Figure S1. Boxplots of antimicrobial resistance gene (ARG) abundance and integron gene abundance in log_10_ gene copies per liter (gc/L) of water for 2018 samples across all sites (n=22).

**
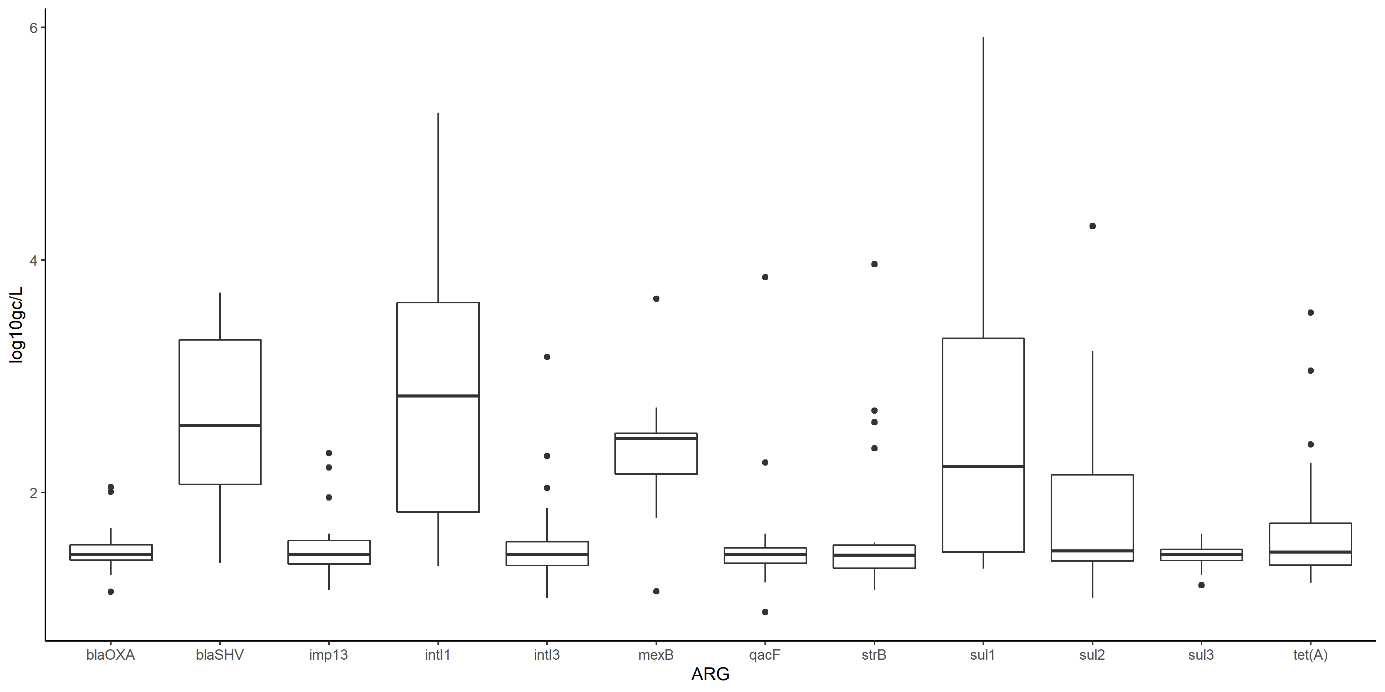
**

Figure S2. Boxplots of antimicrobial resistance gene (ARG) abundance and integron gene abundance in log_10_ gene copies per liter (gc/L) of water for 2019 samples across all sites (n=10).


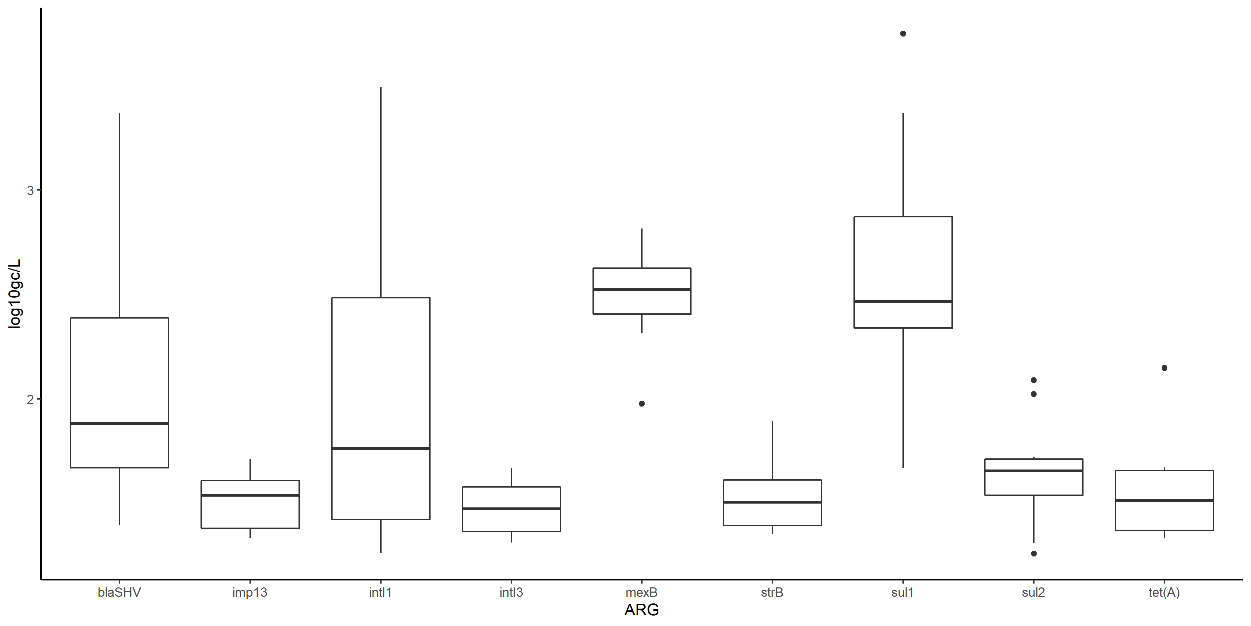


Figure S3. Boxplots of antimicrobial resistance gene (ARG) abundance and integron gene abundance in log_10_ gene copies per gram (gc/g) of sediment for 2018 samples across all sites (n=20).


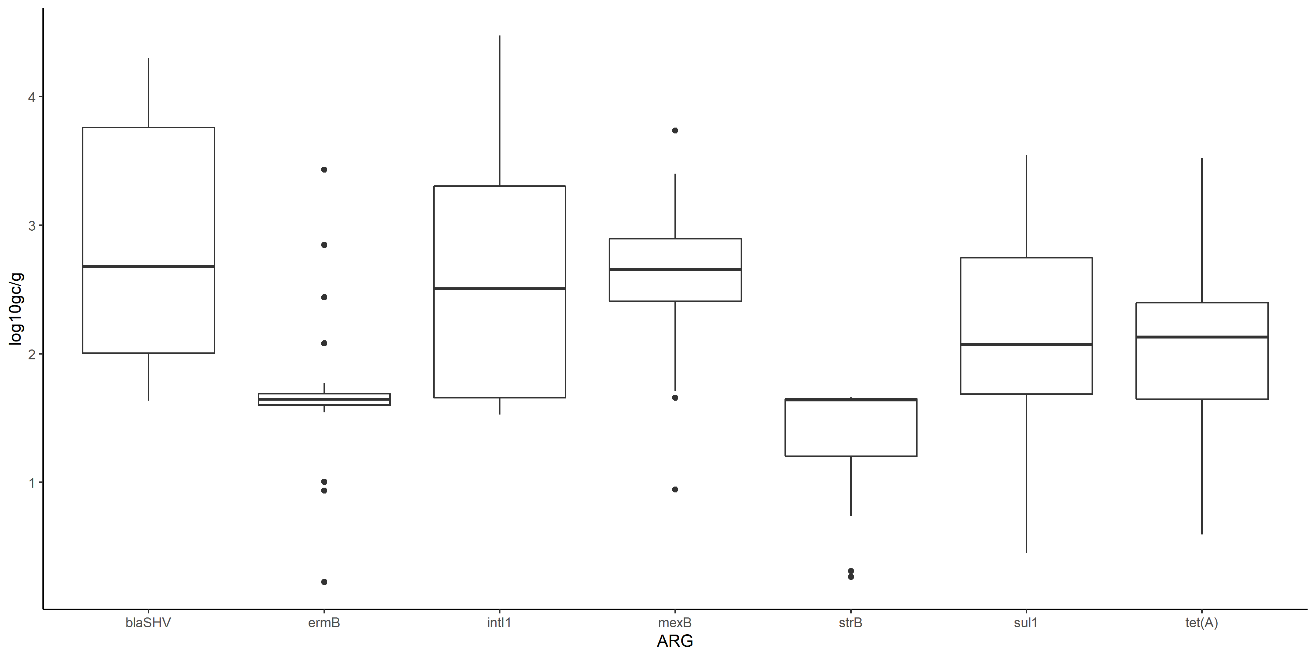


Figure S4. Boxplots of antimicrobial resistance gene (ARG) abundance and integron gene abundance in log_10_ gene copies per gram (gc/g) of sediment for 2019 samples across all sites (n=12).


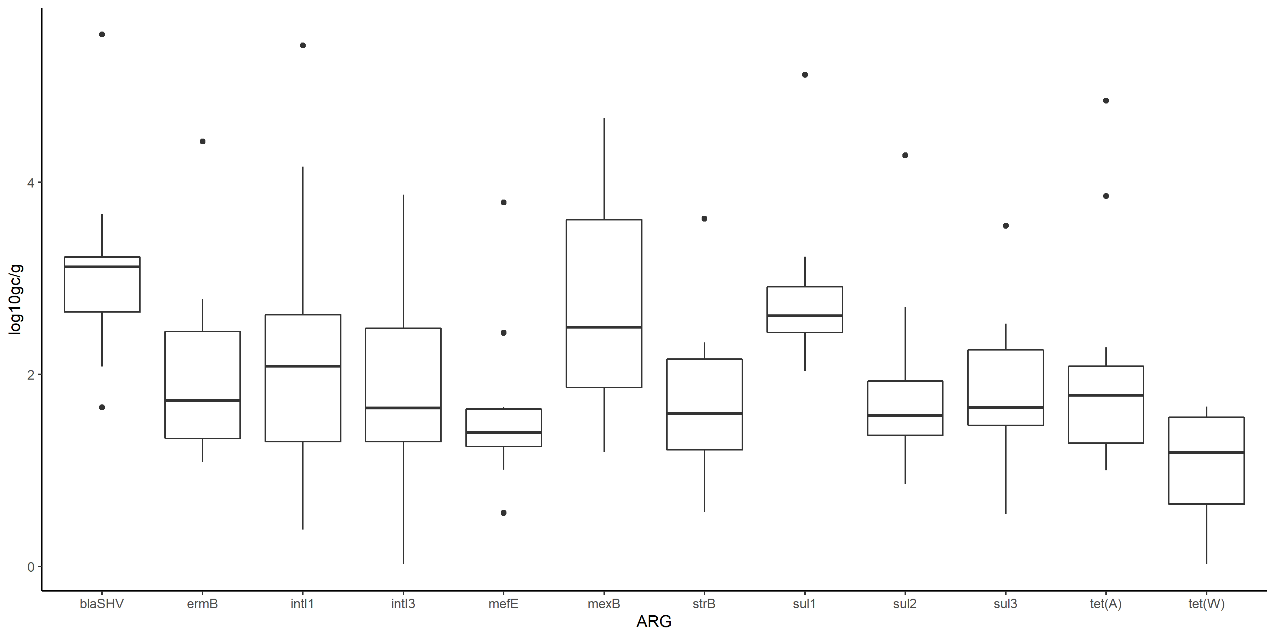


Figure S5. Estimated marginal mean (EMM) plot (bars represent standard errors) for log_10_ *sul1* abundance in water samples in 2019 resulting from the linear mixed model when adjusting for wastewater treatment plant.


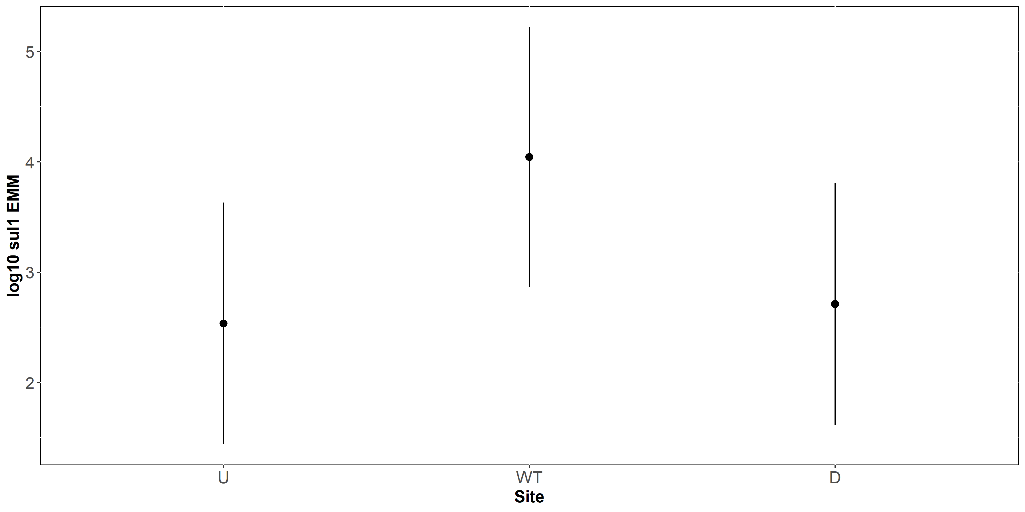


U: Upstream, D: Downstream, WT: Wastewater treatment plant.

Figure S6. Estimated marginal mean (EMM) plot (bars represent standard errors) for log_10_ *sul2* abundance in water samples in 2019 resulting from the linear mixed model when adjusting for wastewater treatment plant.


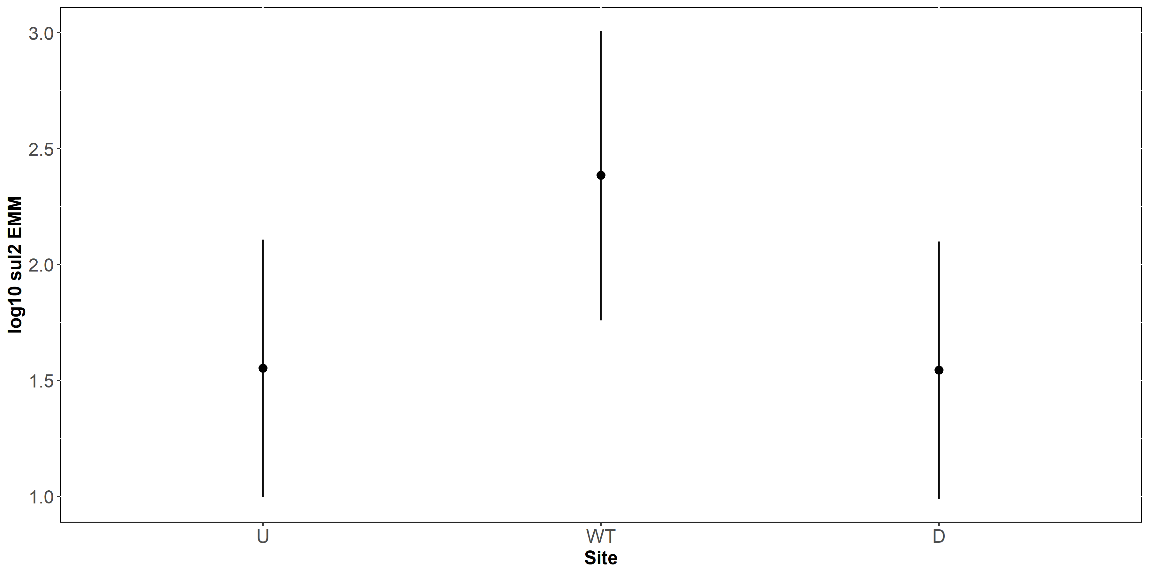


U: Upstream, D: Downstream, WT: Wastewater treatment plant.

Figure S7. Estimated marginal mean (EMM) plot (bars represent standard errors) for log_10_ *bla*_SHV_ abundance in sediment samples in 2019 resulting from the linear mixed model when adjusting for wastewater treatment plant.


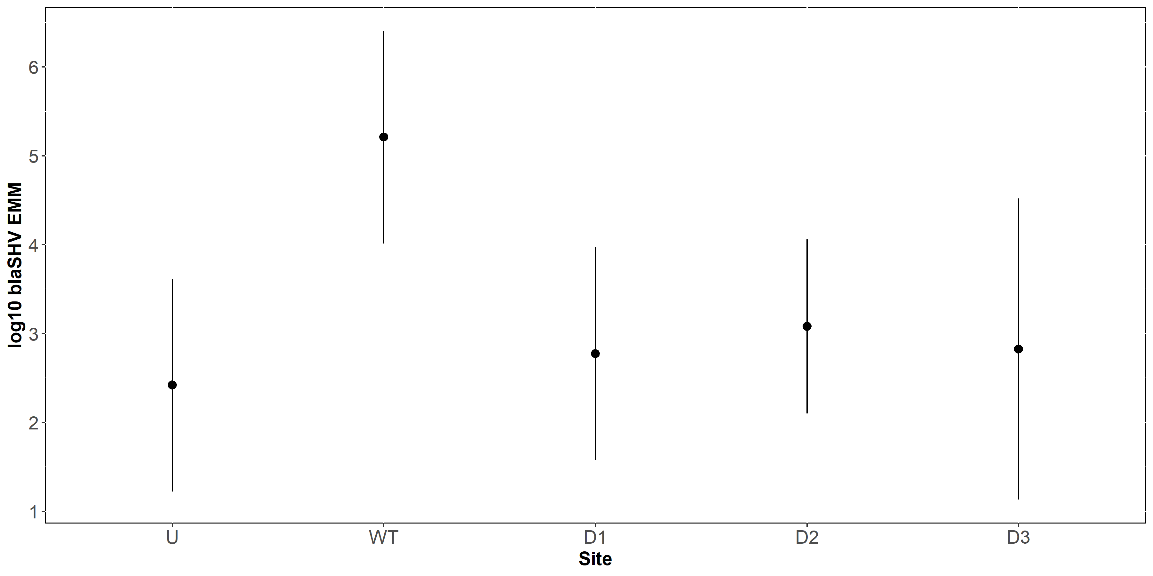


U: Upstream, D1: Downstream site 1, D2: Downstream site 2; D3: Downstream site 3; WT: Wastewater treatment plant.

Figure S8. Estimated marginal mean (EMM) plot (bars represent standard errors) for log_10_ *intI1* abundance in sediment samples in 2019 resulting from the linear mixed model when adjusting for wastewater treatment plant.


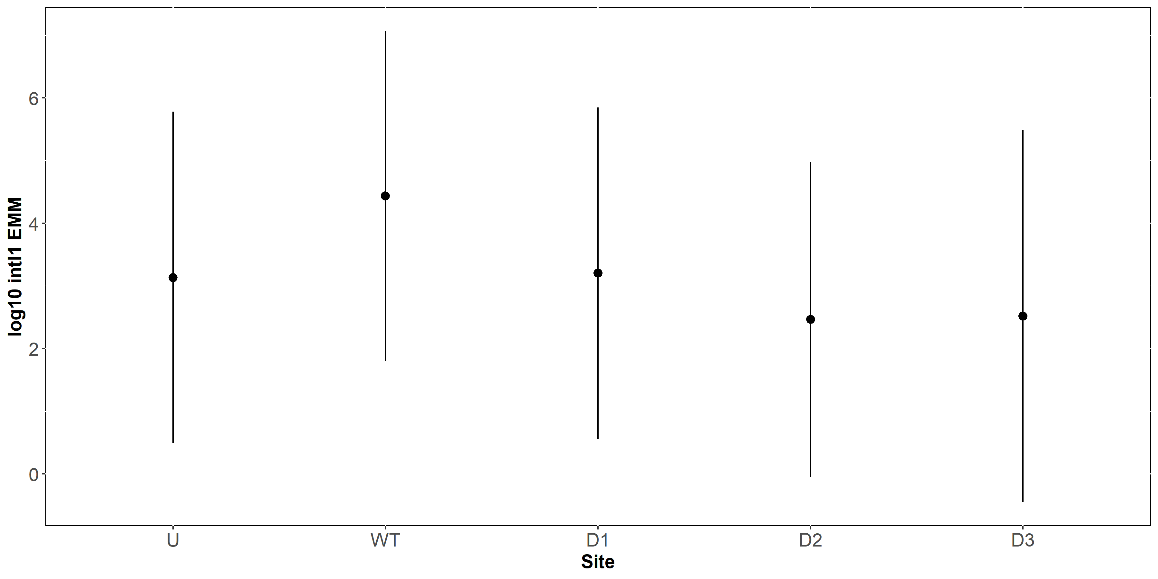


U: Upstream, D1: Downstream site 1, D2: Downstream site 2;

D3: Downstream site 3; WT: Wastewater treatment plant.

Figure. S9. Estimated marginal means (EMM) plot (bars represent standard errors) for log_10_ *mexB* abundance in sediment samples in 2019 resulting from the linear mixed model when adjusting for wastewater treatment plant.


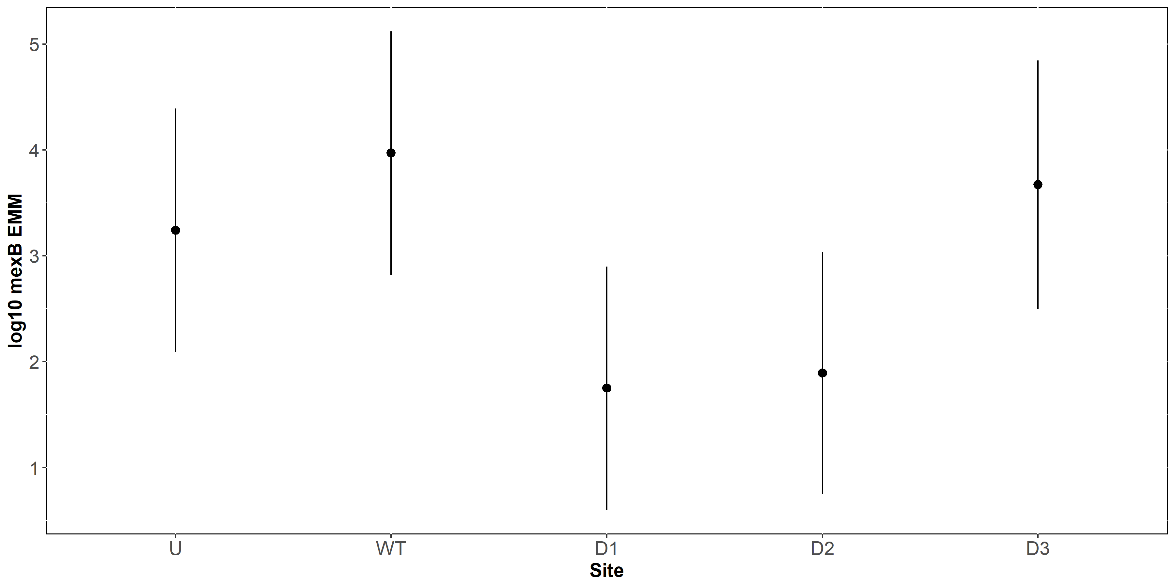


U: Upstream, D1: Downstream site 1, D2: Downstream site 2;

D3: Downstream site 3; WT: Wastewater treatment plant.

Figure. S10. Estimated marginal means (EMM) plot (bars represent standard errors) for log_10_ *sul2* abundance in sediment samples in 2019 resulting from the linear mixed model when adjusting for wastewater treatment plant.


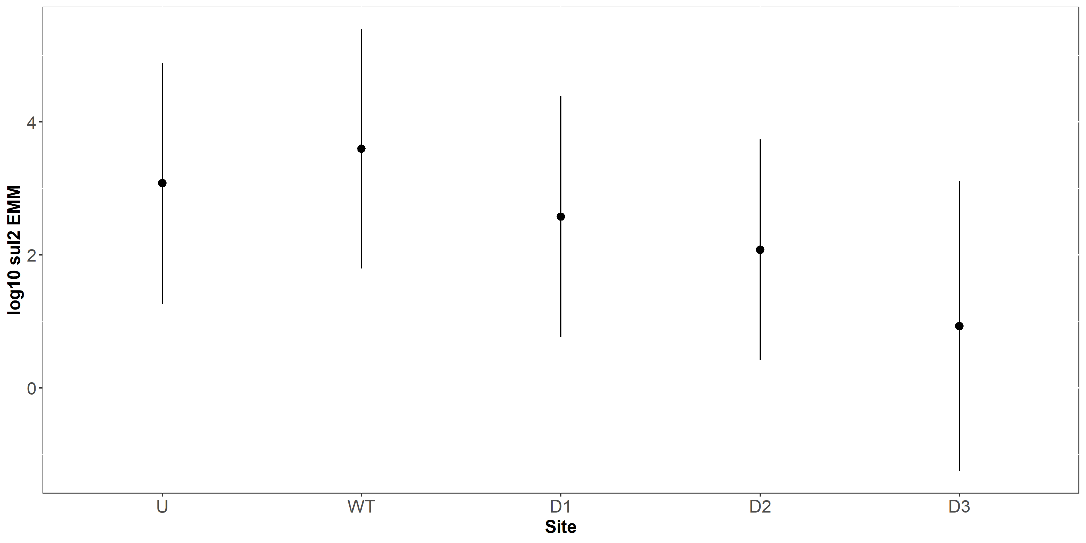


U: Upstream, D1: Downstream site 1, D2: Downstream site 2;

D3: Downstream site 3; WT: Wastewater treatment plant.

Figure S11. Estimated marginal means (EMM) plot (bars represent standard errors) for log_10_ *bla*_SHV_ abundance in water 2018 resulting from the linear mixed model when adjusting for waterbody site.


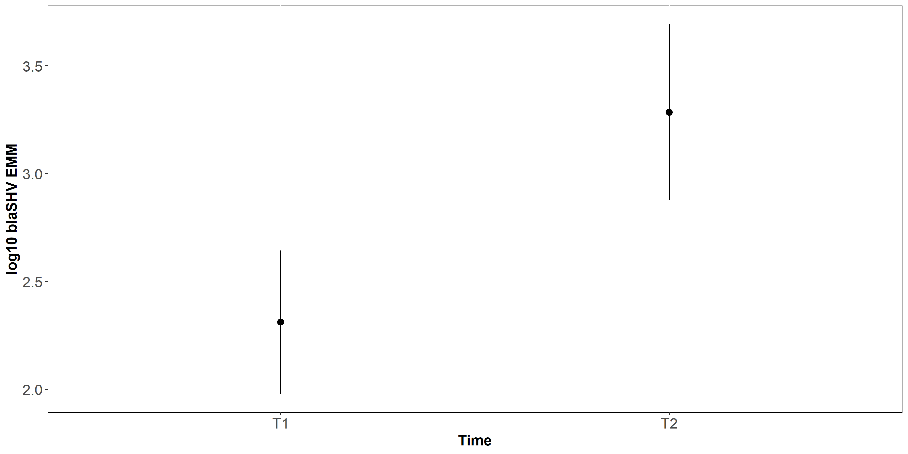


T1: Time 1. T2: Time2.

Figure S12. Estimated marginal means (EMM) plot (bars represent standard errors) for log_10_ *intI1* abundance in water 2018 resulting from the linear mixed model when adjusting for waterbody site.


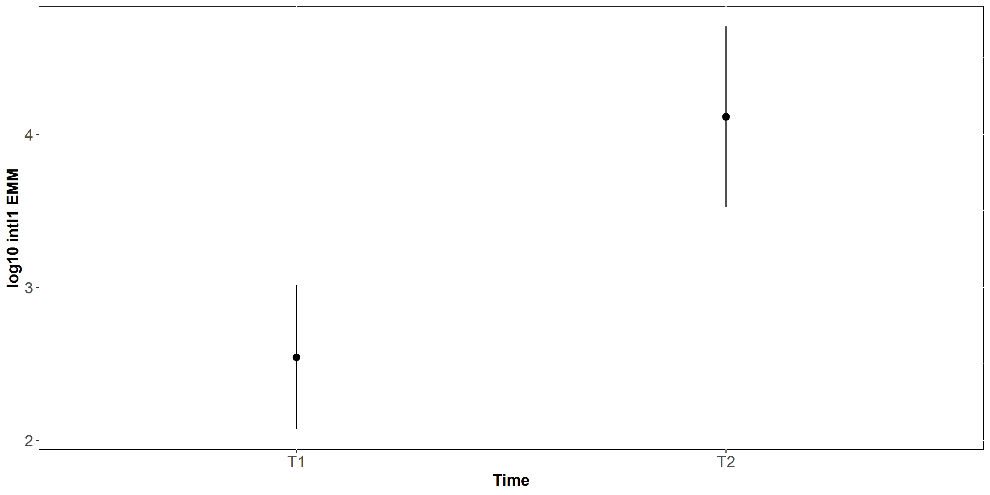


T1: Time 1. T2: Time2.

**Supplementary References**

1 Smith, C. M. & Hill, V. R. Dead-end hollow-fiber ultrafiltration for recovery of diverse microbes from water. *Appl. Environ. Microbiol.* **75**, 5284 (2009). doi:10.1128/AEM.00456-09.

2 Kerrigan, J. F., Sandberg, K. D., Engstrom, D. R., LaPara, T. M. & Arnold, W. A. Sedimentary record of antibiotic accumulation in Minnesota Lakes. *Sci. Total Environ.* **621**, 970-979 (2018). https://doi.org/10.1016/j.scitotenv.2019.07.092.

3 Muyzer, G., de Waal, E. C. & Uitterlinden, A. G. Profiling of complex microbial populations by denaturing gradient gel electrophoresis analysis of polymerase chain reaction-amplified genes coding for 16S rRNA. *Appl. Environ. Microbiol*. **59**, 695 (1993).

4 Szczepanowski, R. *et al.* Detection of 140 clinically relevant antibiotic-resistance genes in the plasmid metagenome of wastewater treatment plant bacteria showing reduced susceptibility to selected antibiotics. *Microbiology.* **155**, 2306-2319 (2009). doi:https://doi.org/10.1099/mic.0.028233-0.

5 Ross, J. & Topp, E. Abundance of antibiotic resistance genes in bacteriophage following soil fertilization with dairy manure or municipal biosolids, and evidence for potential transduction. *Appl. Environ. Microbiol*. **81**, 7905 (2015). doi:10.1128/AEM.02363-15.

6 Chia, J.-H. *et al.* Development of a multiplex PCR and SHV melting-curve mutation detection system for detection of some SHV and CTX-M β-lactamases of *Escherichia coli*, *Klebsiella pneumoniae*, and *Enterobacter cloacae* in Taiwan. *J. Clin. Microbiol.* **43**, 4486-4491 (2005). doi: 10.1128/JCM.43.9.4486-4491.2005.

7 Chen, J., Yu, Z., Michel, F. C., Wittum, T. & Morrison, M. Development and application of real-time PCR assays for quantification of genes conferring resistance to macrolides-lincosamides-streptogramin B in livestock manure and manure management systems. *Appl. Environ. Microbiol*. **73**, 4407 (2007). doi:10.1128/AEM.02799-06.

8 Ma, Y. *et al.* Effect of various sludge digestion conditions on sulfonamide, macrolide, and tetracycline resistance genes and class I integrons. *Environ. Sci. Technol.* **45**, 7855-7861 (2011). doi:10.1021/es200827t.

9 Goldstein, C. *et al.* Incidence of class 1 and 2 integrases in clinical and commensal bacteria from livestock, companion animals, and exotics. *Antimicrob. Agents. Chemother.* **45**, 723 (2001). doi:10.1128/AAC.45.3.723-726.2001.

10 Barraud, O., Baclet, M. C., Denis, F. & Ploy, M. C. Quantitative multiplex real-time PCR for detecting class 1, 2 and 3 integrons. *J. Antimicrob. Chemother*. 1642-1645 (2010). doi:10.1093/jac/dkq167.

11 McNamara, P. J., LaPara, T. M. & Novak, P. J. The Impacts of triclosan on anaerobic community structures, function, and antimicrobial resistance *Environ. Sci. Technol.* **48**, 7393-7400 (2014). doi:10.1021/es501388v.

12 Cummings, D. E. *et al.* Broad dissemination of plasmid-mediated quinolone resistance genes in sediments of two urban coastal wetlands. *Environ. Sci. Technol.* **45**, 447-454, (2011). doi:10.1021/es1029206.

13 Heuer, H. & Smalla, K. Manure and sulfadiazine synergistically increased bacterial antibiotic resistance in soil over at least two months. *Environ. Microbiol.* **9**, 657-666 (2007). doi:https://doi.org/10.1111/j.1462-2920.2006.01185.x.

14 Pei, R., Kim, S.-C., Carlson, K. H. & Pruden, A. Effect of river landscape on the sediment concentrations of antibiotics and corresponding antibiotic resistance genes (ARG). *Water Res.* **40**, 2427-2435 (2006). doi:https://doi.org/10.1016/j.watres.2006.04.017.

15 Ng, L. K., Martin, I., Alfa, M. & Mulvey, M. Multiplex PCR for the detection of tetracycline resistant genes. *Mol. Cell. Probes.* **15**, 209-215 (2001). doi:https://doi.org/10.1006/mcpr.2001.0363.

16 Aminov, R. I., Garrigues-Jeanjean, N. & Mackie, R. I. Molecular ecology of tetracycline resistance: development and validation of primers for detection of tetracycline resistance genes encoding ribosomal protection proteins. *Appl. Environ. Microbiol*. **67**, 22 (2001). doi:10.1128/AEM.67.1.22-32.2001.

17 Ghosh, S., Sadowsky, M. J., Roberts, M. C., Gralnick, J. A. & LaPara, T. M. *Sphingobacterium sp*. strain PM2-P1-29 harbours a functional tet(X) gene encoding for the degradation of tetracycline. *J. Appl. Microbiol.* **106**, 1336-1342 (2009). doi:https://doi.org/10.1111/j.1365-2672.2008.04101.x.

18 Bell, J. M., Paton, J. C. & Turnidge, J. Emergence of vancomycin-resistant *Enterococci* in Australia: phenotypic and genotypic characteristics of isolates. *J. Clin. Microbiol.* **36**, 2187 (1998). doi:10.1128/JCM.36.8.2187-2190.1998.

19 USEPA. National pollutant discharge elimination system permit regulation and effluent limitation guidelines and standards for concentrated animal feeding operations (CAFOs); final rule. *Fed. Regist.* **68**, 7176-7274. <https://www.federalregister.gov/documents/2003/02/12/03-3074/national-pollutant-discharge-elimination-system-permit-regulation-and-effluent-limitation-guidelines> (2003).
